# Impaired neuron differentiation in GBA-associated Parkinson’s disease is linked to cell cycle defects in organoids

**DOI:** 10.1038/s41531-023-00616-8

**Published:** 2023-12-18

**Authors:** Isabel Rosety, Alise Zagare, Claudia Saraiva, Sarah Nickels, Paul Antony, Catarina Almeida, Enrico Glaab, Rashi Halder, Sergiy Velychko, Thomas Rauen, Hans R. Schöler, Silvia Bolognin, Thomas Sauter, Javier Jarazo, Rejko Krüger, Jens C. Schwamborn

**Affiliations:** 1 Developmental and Cellular Biology, Luxembourg Centre for Systems Biomedicine (LCSB), University of Luxembourg, Esch-sur-Alzette, Luxembourg; 2 OrganoTherapeutics SARL-S, Esch-sur-Alzette, Luxembourg; 3 Translational Neuroscience, Luxembourg Centre for Systems Biomedicine, University of Luxembourg, Esch-sur-Alzette, Luxembourg; 4 Biomedical Data Science group, Luxembourg Centre for Systems Biomedicine, University of Luxembourg, Esch-sur-Alzette, Luxembourg; 5 Systems Ecology Group, Luxembourg Centre for Systems Biomedicine, University of Luxembourg, Esch-sur-Alzette, Luxembourg; 6 Max Planck Institute for Molecular Biomedicine, MPG White Paper Group - Animal Testing in the Max Planck Society, Muenster, Germany; 7 Department of Life Sciences and Medicine, University of Luxembourg, Belvaux, 4367 Luxembourg; 8 Transversial Translational Medicine, Luxembourg Institute of Health (LIH), 1 A-B rue Thomas Ediison, L-1445 Strassen, Luxembourg

**Keywords:** Cellular neuroscience, Parkinson's disease, Neural stem cells

## Abstract

The mechanisms underlying Parkinson’s disease (PD) etiology are only partially understood despite intensive research conducted in the field. Recent evidence suggests that early neurodevelopmental defects might play a role in cellular susceptibility to neurodegeneration. To study the early developmental contribution of GBA mutations in PD we used patient-derived iPSCs carrying a heterozygous N370S mutation in the GBA gene. Patient-specific midbrain organoids displayed GBA-PD relevant phenotypes such as reduction of GCase activity, autophagy impairment, and mitochondrial dysfunction. Genome-scale metabolic (GEM) modeling predicted changes in lipid metabolism which were validated with lipidomics analysis, showing significant differences in the lipidome of GBA-PD. In addition, patient-specific midbrain organoids exhibited a decrease in the number and complexity of dopaminergic neurons. This was accompanied by an increase in the neural progenitor population showing signs of oxidative stress-induced damage and premature cellular senescence. These results provide insights into how GBA mutations may lead to neurodevelopmental defects thereby predisposing to PD pathology.

## Introduction

Parkinson’s disease (PD) is considered the second most common neurodegenerative disorder. It is characterized by the loss of dopaminergic (DA) neurons in the substantia nigra pars compacta of the midbrain. Heterozygous mutations in the GBA1 gene represent the most common genetic risk factor for PD^1^. The GBA1 gene encodes the lysosomal enzyme β-glucocerebrosidase (GCase), which catalyzes the hydrolysis of glucosylceramide (GlcCer) into ceramide and glucose^2^. Homozygous GBA mutations cause Gaucher disease (GD), the most prevalent lysosomal storage disorder, which is characterized by the accumulation of GlcCer in different tissues^3^. However, the underlying molecular mechanisms through which mutant GCase leads to PD is not yet fully understood. Both toxic gain of and loss of GCase function have been proposed^4^, although the two hypotheses are not mutually exclusive.

PD is classically considered an age-related disorder. However, increasing evidence suggests that neurodevelopmental defects might increase the susceptibility to develop PD^5–8^. Neurodevelopmental alterations such as impaired midbrain dopaminergic (mDA) neuron differentiation or metabolic perturbations could affect the viability and resistance to insults and stressors of adult mDA neurons. Interestingly, recent reports have associated PD with metabolic alterations^9–11^. In addition, Gaucher disease (GD) patients, who carry a homozygous mutation in the GBA1 gene, are known to experience metabolic disturbances^12^. This led to the hypothesis that the GBA-N370S heterozygous mutation may result in metabolic changes that could have an impact in neurodevelopment.

Human induced pluripotent stem cell (iPSC) technology has allowed the opportunity to investigate the role of genetic mutations in the pathogenesis of PD. A recent breakthrough was achieved in the field with the derivation of human midbrain organoids (MOs) from iPSCs. This allowed the generation of 3D human tissue in vitro that recapitulates the physiology and complexity of the human brain^13^. Moreover, midbrain organoids derived from iPSCs mimic early embryonic neurodevelopment^14^, thereby constituting an ideal model to investigate PD-related neurodevelopmental phenotypes.

In this study, we generated midbrain organoids from patients carrying a heterozygous GBA-N370S mutation. We exploited gene expression data to reconstruct a context-specific metabolic model in order to generate predictions which were validated with experimental data. The main metabolic subsystems deregulated as a result of the GBA-N370S mutation were the sphingolipid pathway and metabolite extracellular transport reactions. Moreover, we showed that GBA-PD organoids displayed relevant disease phenotypes such as reduced GCase enzymatic activity as well as mitochondrial and autophagic dysfunction. Our results suggest that the heterozygous GBA-N370S mutation has a negative effect in the differentiation of neural precursors to neurons. These data underline the early pathogenic contribution of GCase function and may explain the selective mDA neuronal vulnerability in GBA-PD.

## Results

### Generation and characterization of GBA-PD patient-specific MOs

**Fig. 1:**
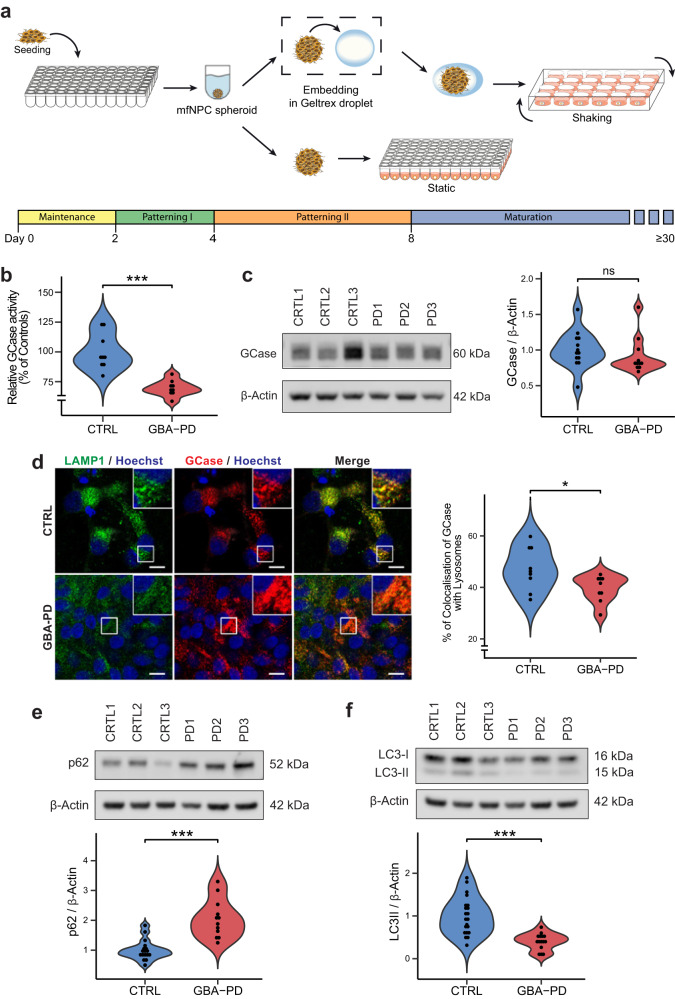
Generation and characterization GBA-PD midbrain organoids. **a** Schematic overview of the protocol used for the generation of midbrain organoids. mfNPCs, floorplate neural precursors. **b** GCase enzyme activity in differentiated MO cultures was significantly decreased (40% reduction) when compared with controls. The data represent a summary of three independent differentiation experiments per line each analyzed in triplicate at DIV30. Values are normalized to the average of controls per experiment. Wilcoxon T-test; ***p < 0.001. **c** GCase protein levels are not altered at DIV30. Representative western blot analysis and respective quantification. The data represent a summary of at least three independent differentiation experiments per line each analyzed in triplicate. Values are normalized to the average of controls per experiment. **d** Decreased percentage of colocalization of GCase with lysosomes in GBA-PD organoids at DIV30. GCase (red), LAMP1 (green). Representative confocal images with their respective zoomed region of interest (ROI) and their quantification (scale bar, 10 μm). The data represent a summary of three independent differentiation experiments for all cell lines, normalized to the average of controls per experiment. Wilcoxon T-test; *p < 0.05. **e**, **f** Representative western blot and quantification of expression of the autophagy markers p62 and LC3 at DIV30. Data represents a summary of at least five independent differentiation experiments. Values are normalized to the average of controls per experiment. Wilcoxon T-test; ***p < 0.001.

To investigate whether mutant GCase is linked to an altered metabolism leading to neurodevelopmental defects, iPSCs derived from 3 healthy controls (CTRL1, CTRL2, and CTRL3) and 3 GBA-PD patients (PD1, PD2, and PD3) were used to generate midbrain organoid models (Supplementary Table 1). All iPSC lines displayed embryonic stem cell-like morphology and expressed the pluripotency markers SOX2, TRA-1-81, TRA-1-60, OCT4, SSEA4, and NANOG, as well as presenting a normal karyotype. They were subsequently screened for the GBA-N370S heterozygous mutation, which was absent in all the healthy controls and was confirmed in all three GBA-PD cell lines. In addition, the LRRK2-G2019S mutation was discovered in the patient PD3 (Full iPSC characterization available in “Data availability”). Human iPSCs were differentiated into midbrain floorplate neural precursors (mfNPCs) as described before^15^. The successful derivation of mfNPCs was assessed by confirming the expression of the neural stem cell markers Nestin and SOX2 along with the absence of the dorsal marker Pax6 (Supplementary Fig. 1a, b). Next, we generated midbrain organoids (MOs) from the mfNPCs based on a protocol previously described^16^. Organoids were either embedded in a droplet of geltrex or kept unembedded in a low-attachment 96-well plate (Fig. 1a, Supplementary Fig. 2), depending on the intended downstream assays.

We then sought to confirm the decrease of GCase activity that is associated with the N370S mutation. As expected, the enzyme activity of GCase in GBA-PD MOs was significantly reduced (Fig. 1b). No differences were found in the levels of GCase protein at 30 days of in vitro organoid differentiation (DIV) (Fig. 1c), but they were significantly decreased at DIV60 (Supplementary Fig. 3a). Moreover, mutations in the GBA gene are known to cause GCase retention in the ER, leading to reduced levels of the enzyme in the lysosomes^17,18^. Therefore, we measured the amount of GCase protein found in the lysosomal compartment by colocalization analysis with the lysosomal marker LAMP1 and found a significant reduction of the enzyme located in lysosomes (Fig. 1d).

GCase deficiency has been shown to impair the lysosomal degradation capacity of dopaminergic neurons^18^, we therefore examined the glycohydrolase activity of the lysosomal enzymes β-hexosaminidase, β-galactocerebrosidase and β-galactosidase. No significant differences were observed in β-hexosaminidase or β-galactocerebrosidase activity of GBA-PD MOs compared to unaffected controls, however, an increase in β-galactosidase activity was detected in patient-derived MOs (Supplementary Fig. 3b). To further investigate a possible impairment of the lysosomal degradation capacity, we examined the autophagy markers p62/SQSTM1 and LC3 to assess autophagy flux. We found that p62 levels were increased in GBA-PD MOs under basal conditions (Fig. 1e) as previously shown^18^, whereas LC3-II levels as well as LC3-II/-LC3 I ratio were decreased (Fig. 1f, Supplementary Fig. 3c), suggesting that the formation of autophagosomes might be impaired.

Mitochondrial dysfunction has been proposed as a key mechanism in the pathogenesis of PD and has been reported in GBA-PD cellular and animal models^19–21^. We investigated mitochondrial mass by quantification of the mitochondrial membrane proteins VDAC and TOM20. Whereas we found no differences in TOM20 levels (Supplementary Fig. 3d), there was a significant decrease in VDAC (Supplementary Fig. 3e), probably indicating that while mitochondrial mass is unaffected in GBA-PD MOs, their content in the voltage-dependent anion channel, VDAC, is reduced. Based on this, we speculated that mitochondria functionality might be impaired. In order to evaluate this, we measured mitochondrial oxygen consumption rates (OCRs); indeed, GBA-PD organoids displayed significantly reduced mitochondrial ATP as well as decreased coupling efficiency, and higher proton leak compared to unaffected controls (Supplementary Fig. 3f), which clearly indicates that mitochondrial functionality is compromised.

In summary, patient-specific midbrain organoids exhibit characteristic pathological features of GBA-associated PD, such as impaired GCase activity, reduced lysosomal content of GCase, impaired autophagy flux and mitochondrial phenotypes. All together this qualifies this model as an excellent tool for further investigations.

### Computational modeling shows that the GBA-N370S mutation has a high impact on cellular metabolism

After the establishment and validation of a midbrain PD model consistent with GBA-PD-associated phenotypes, we addressed the effect of the heterozygous GBA-N370S mutation on metabolic changes. Genome-scale metabolic (GEM) models are an in silico tool for the identification of metabolic flux alterations underlying disease phenotypes and has proven to be useful to generate accurate predictions and relevant hypothesis for metabolism research^22,23^. Here, we use one of the most comprehensive human metabolic models, Recon3^24^ to reconstruct context-specific models of healthy and GBA-PD midbrain organoids. RNA-seq analysis was conducted on DIV30 midbrain organoids from the CTRL1, CTRL2, PD1, and PD2 cell lines and was used to build cell line-specific models with rFASTCORMICS^25^ capturing GBA-N370S associated metabolic alterations.

**Fig. 2:**
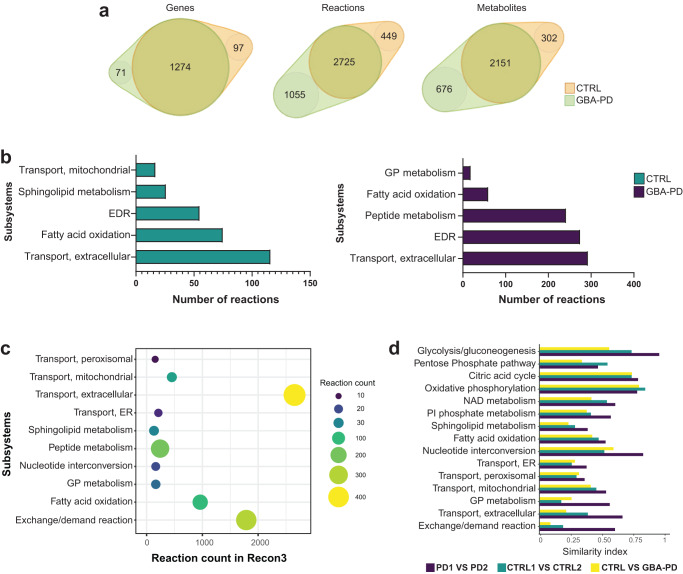
Metabolic modeling of GBA-PD. **a** Venn diagram representation of the structural model analysis. **b** Comparison of model composition by reactions. Reactions found exclusively in control models (green) or in GBA-PD (purple) were mapped to the Recon 3 subsystems, and the top five most different subsystems based on the reaction number are listed for each condition. EDR = exchange/demand reaction, GP = glycerophospholipid. **c** The top 10 most different subsystems between control (CTRL1 and CTRL2) and GBA-PD (PD1 and PD2) models after pooling the exclusive reactions of the two conditions. The size and color of the dot represent the number of reactions per subsystem. The location in relation to the x-axis represents the size of the subsystem in the generic Recon3. Subsystems are positioned on the y-axis in alphabetical order. GP = glycerophospholipid, ER = endoplasmic reticulum. **d** Similarity index of flux variability analysis optimizing for ATP demand for subsystems of interest. SI between CTRL1 vs CTRL2 and SI between PD1 and PD2 models were compared, as well as the SI between the CTRL (CTRL1 and CTRL2) and GBA-PD (PD1 and PD2). SI of 0 represents a complete mismatch in flux variability between the models, whereas a SI of 1 represents the highest similarity in flux variability. PI = Phosphatidylinositol, ER = endoplasmic reticulum, GP = glycerophospholipid.

First, we performed structural model analysis to identify differences between the control and GBA-PD models in gene, metabolite, and reaction composition (Fig. 2a and Supplementary Table 3). We identified 97 genes, 449 reactions, and 302 unique metabolites present only in the control models but not in patient-derived models. In contrast, GBA-PD models shared 71 genes, 1055 reactions, and 676 unique metabolites that were absent in the control models. Next, we performed gene enrichment analysis for the control and patient model unique genes (Supplementary Fig. 4a). We found that the most enriched biological processes of control-specific genes were associated to lipid metabolism, transmembrane transport, and polyol transport, while GBA-PD model-specific genes showed a particular enrichment in the glycosylation and carbohydrate metabolic processes. Further, we analyzed the Recon3 subsystems of the control and patient-specific reactions (Fig. 2b). Among the top five subsystems containing the highest number of control-specific reactions we identified mitochondrial transport, sphingolipid metabolism, extracellular transport, and fatty acid oxidation. The highest number of reactions specific to the GBA-PD models belong to glycerophospholipid metabolism, fatty acid oxidation, peptide metabolism, and extracellular transport (Fig. 2b). Importantly, within the extracellular metabolite transport reactions, dopamine extracellular transport belongs to the subset of reactions present only in control models and was not found in the GBA-PD models (Supplementary Table 4). Next, we pooled the count of control- and mutant-specific reactions per subsystem in order to identify metabolic pathways with the most different reaction composition between the two conditions (Fig. 2c, Supplementary Table 5). The subsystems with the most different reactions were extracellular metabolite transport, exchange/demand reactions, and peptide metabolism, followed by fatty acid oxidation, sphingolipid metabolism, and glycerophospholipid metabolism. We then performed flux variability analysis (FVA) on these subsystems optimizing for ATP production to mimic a situation of high energetic demand similar to the physiological state of a mDA neuron^26^. We included in our analysis the central energy pathways as there is evidence that these pathways could be dysregulated^27^. For the subsystems of interest, we then compared flux distribution similarity based on FVA between mutants and controls using a similarity index (SI) (Fig. 2d). In addition, we computed the SI between both control models and between both mutant models separately (Fig. 2d). This allowed us to assess the variability between cell lines of the same condition. We found that in a situation of high ATP demand, the most different flux distribution was observed in exchange/demand reactions and extracellular transport, followed by sphingolipid and glycerophospholipid metabolism. However, when considering the subsystems with a relative low variability within the same condition, the biggest differences between patients and controls were observed in the glycolysis pathway, after exchange/demand reactions and extracellular metabolite transport.

Interestingly, we observed that the SI between both mutant models were, in general, higher than between both control models for all the metabolic pathways of interest. This indicates that the variability within the GBA-PD condition is lower than in the controls, suggesting that the GBA-N370S mutation has a substantial effect on metabolism.

Overall, these findings suggest that lipid metabolism in GBA-PD organoids could be largely affected, along with important disturbances in the extracellular transport.

### Lipid alterations in PD-N370S midbrain organoids

**Fig. 3:**
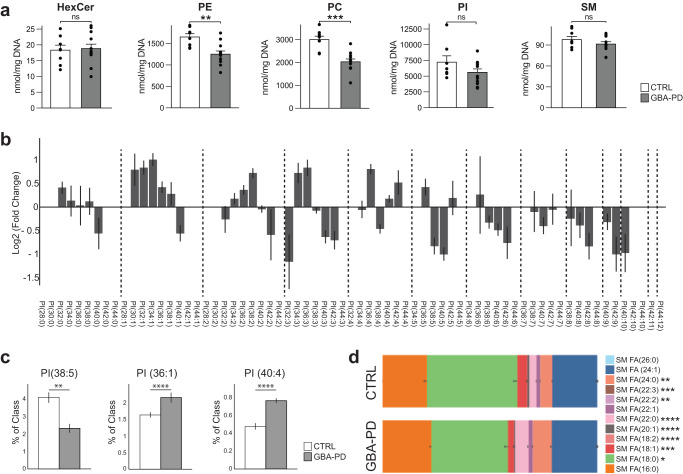
Lipidomics analysis showing differences in lipid classes and lipid species composition. **a** Differences in phosphatidylethanolamine (PE) and phosphatidylcholine (PC) levels but not hexosylceramides (HexCer), sphingomyelins (SM) or phosphatidylinositol (PI) in HILIC LC-MS/MS based lipidomic analysis of DIV30 organoids. Data represent a summary of four independent organoid differentiations, Mann–Whitney U test. **p < 0.01, ****p < 0.0001. Error bars represent standard error of the mean. **b** Pairwise comparison between GBA-PD vs control of phosphatidylinositol (PI) lipid species denoted by their sum notation. Data expressed as the Log2 of the fold change. Error bars represent standard error of the mean. **c** Plots for PI molecular species PI (38:5), PI (36:1), PI (40:4), each plot corresponds to the most enriched species for that sum notation: (18:1/20:4), PI (18:0/18:1), and PI (18:0/22:4), respectively. Data represent a summary of four independent organoid differentiations and is expressed as the percentage of the entire PI class. *p*-values were calculated using a one-way ANOVA test. FDR adjusted *p*-values were calculated using the Benjamini–Hochberg procedure. **p < 0.01, ****p < 0.0001. Error bars represent standard error of the mean. **d** Fatty acid composition of the lipid species within the sphingomyelin lipid class. Data represent a summary of four independent organoid differentiations expressed as relative quantification, where each species is normalized to the total amount of lipid of its class. *p*-values were calculated using a one-way ANOVA test. FDR adjusted *p*-values were calculated using the Benjamini–Hochberg procedure. *p < 0.05, **p < 0.01, ***p < 0.001, ****p < 0.0001. Error bars represent standard error of the mean.

Among the predicted metabolic changes, glycerophospholipid metabolism and sphingolipid metabolism were recurrently highlighted pathways (Fig. 2b–d), implying that lipid metabolism in GBA-PD organoids could be largely affected. In order to confirm that, we performed a comprehensive lipidomic analysis including over 2000 lipid species across 16 different lipid classes. We visualized the results using the t-SNE dimensionality reduction technique and observed that the lipidomics data from GBA-N370s MOs and controls formed two clearly separated distinct clusters (Supplementary Fig. 4b), respectively, suggesting a dramatic difference in the lipidomes between the two conditions. We further scrutinized these results by analyzing the levels of hexosylceramides (HexCer), which comprise glucosylceramide (GlcCer), the primary physiological substrate of GCase, as well as galactosylceramide. The predominant HexCer species was C18:0 (Supplementary Fig. 4c) as reported in the human brain^28,29^ and its levels were significantly lower in GBA-PD organoids, whereas HexCer C22:0 was significantly higher (Supplementary Fig. 4c). However, the total HexCer levels where not significantly different in GBA-PD MOs compared to controls (Fig. 3a).

Interestingly, the two most abundant phospholipids in the brain, phosphatidylethanolamine (PE) and phosphatidylcholine (PC)^30^, were both decreased in GBA-PD derived organoids (Fig. 3a), this was accompanied with statistically significant differences in other lipid classes, such as triglycerides (TG), monoglycerides (MG) and phosphatidylglycerol (PG) (Fig. S4d). Even though there were no differences in the total levels of phosphatidylinositol (PI), there was an important dissimilarity in the PI species composition (Fig. 3b, c). A total of 45 lipid species were found to differ significantly in their abundance between the two conditions. A lipidomics study of the human PD brain identified a specific decrease in PI (38:5) accompanied by an increase in PI (36:1) and PI (40:4) in the visual cortex^31^. In agreement with this report, the same differences were detected here (Fig. 3c). PIs are the precursors of phosphoinositides (PIPs), that are signaling molecules required for many important cellular processes. The fatty-acyl chain profiles of PIPs correlate with that of PIs^32^, we therefore assumed that the significant differences observed in PI molecular species would translate to an abnormal PIP profile. Consistently, many phosphoinositide signaling genes expressed in the human fetal brain^33^ were differentially expressed in GBA-PD organoids (Supplementary Fig. 4e).

Surprisingly, we did not detect any differences in total levels of sphingomyelins (Fig. 3a), which are synthesized from ceramide and PC, both of which were significantly reduced in patient-derived MOs. However, their species profile was significantly different between the two conditions in terms of fatty acid composition (Fig. 3d).

Our lipidomics results indicate that GBA-PD organoids present a drastically deregulated lipid profile, including differences in steady-state levels for several lipid classes, as well as significant changes in the molecular species within the same class. Strikingly, they recapitulate lipid composition differences that previously have been described in the brain of human PD patients^31^.

### Impaired Dopaminergic neuronal differentiation in GBA-PD midbrain organoids

**Fig. 4:**
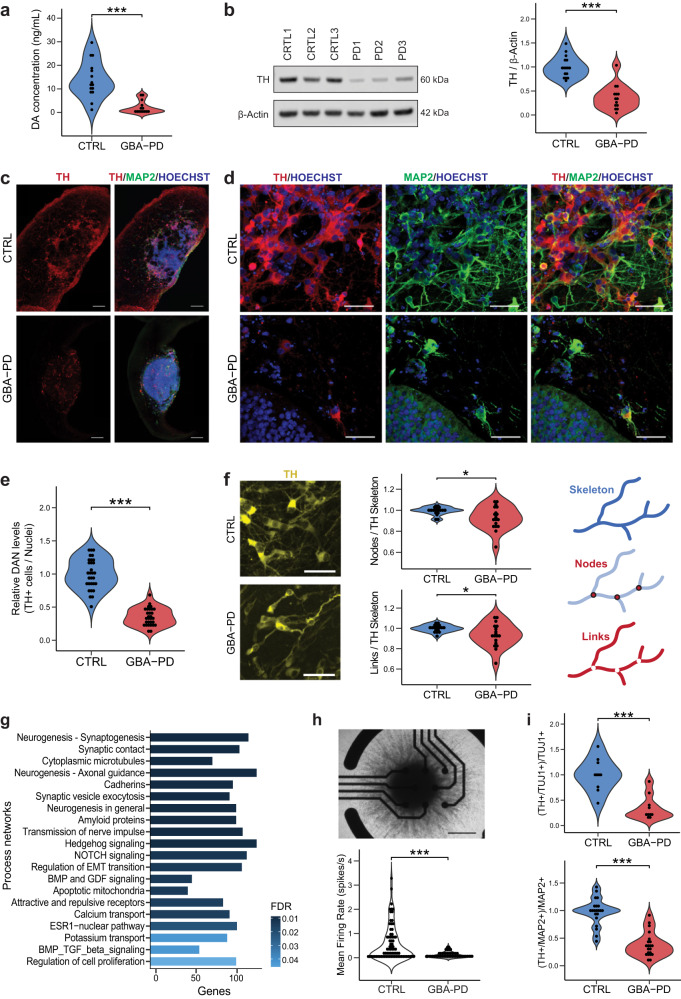
Impaired dopaminergic and general neuronal differentiation in GBA-PD patient-specific midbrain organoids. **a** Levels of extracellular dopamine in culture media at DIV60 were lower for GBA-PD organoids when compared with controls, measured by ELISA. The data represent a summary of five independent differentiation experiments for all cell lines. Wilcoxon T-test; ***p < 0.001. **b** Quantification of TH protein levels and representative western blot at DIV60 showing decreased levels of the protein in heterozygous GBA*-*N370S organoids. Data represents a summary of five independent differentiation experiments normalized to the mean of the controls per batch. Wilcoxon T-test; ***p < 0.001. **c** Representative images of DIV30 midbrain organoids sections stained for TH (red), MAP2 (green), nuclei (blue) (scale bar, 200 μm). **d** Immunofluorescence images of sections from Fig. 3c acquired at 40× (scale bar, 50 μm). **e** High-content automated image analysis of immunofluorescence stainings of dopaminergic neurons in organoids at DIV30 expressed as the proportion of cells expressing TH normalized by total nuclei. Data represents a summary of six independent differentiation experiments normalized to the mean of the controls per batch. Wilcoxon T-test; ***p < 0.001. **f** Neurite branching is less complex in dopaminergic neurons from GBA-PD organoids at DIV30 when compared with controls, measured by the number of nodes (branching points) and links (branches) extracted from the skeletonization of TH mask by the algorithm used for image analysis. Representative immunofluorescence images of TH+ neurons (yellow) showing less complex arborization in GBA-PD condition (scale bar, 50 μm) and graphic illustration of the morphometric features; links, nodes and skeleton. Data is normalized to the mean of the controls per experiment. *n* = 6, Wilcoxon T-test; *p < 0.05. **g** GeneGO MetaCore^TM^ enrichment analysis by process networks showing the top 20 overrepresented processes in DIV30 organoids. **h** Mean firing rate detected by individual electrodes of a multi-electrode array (MEA) system at DIV15 showing that mutant organoids are less electrophysiologically active. The data represent a summary of four independent differentiation experiments for all cell lines. Values are normalized to the mean of the controls per experiment. Wilcoxon T-test; ***p < 0.001. Upper panel shows a representative image of a midbrain organoid positioned on an 8-electrode array in a 96-well tissue culture plate (scale bar, 500 μm). **i** Decreased levels of TH+ cells normalized to the total neuronal population at DIV15 using the early neuronal marker TUJ1 and DIV30 using the late neuronal marker MAP2.

Based on the metabolic modeling, the most affected subsystem is the extracellular transport (Fig. 2b–d), since some of the reactions present exclusively in control models belong to the dopamine extracellular transport (Supplementary Table 4), we sought to experimentally validate whether dopamine release was impaired in GBA-PD organoid cultures. Indeed, we found that dopamine extracellular levels were profoundly decreased in all the MOs with the GBA-N370S mutation compared with control-derived MOs at DIV60 (Fig. 4a). Next, we assessed the levels of tyrosine hydroxylase (TH), the rate-limiting enzyme in the biosynthesis of dopamine, and found that they were equally reduced, not only at DIV60 (Fig. 4b) but also at every time point collected from DIV15 to DIV90 (Supplementary Fig. 5a). To determine whether decreased levels of DA and TH result from a lower enzymatic content in cells or rather a lower number of DA neurons in patient MOs, we quantified the amount of dopaminergic neurons using an automated image analysis algorithm. The proportion of TH+ neurons was significantly decreased (Fig. 4c–e, S5b–d) in GBA-PD organoids compared to controls, at DIV30 and DIV60, but also at earlier timepoints (Supplementary Fig. 5b). Moreover, the neurites of these dopaminergic neurons seemed to be less ramified (Fig. 4f), quantified by the number of links and nodes and this difference becomes even more significant at later timepoints (Supplementary Fig. 5e). In addition to a less complex arborization, the neurites of these dopaminergic neurons presented a higher fragmentation index (Supplementary Fig. 5f), which can be an early sign of neurodegeneration^34^.

We performed an enrichment analysis by process networks on the transcriptomic data obtained from the two control lines (CTRL1 and CTRL2) and the two GBA-PD lines (PD1 and PD2), in order to gain insight into the deregulated processes that might explain the inefficient dopaminergic differentiation in all the GBA-PD organoids (Fig. 4g). Most of the enriched processes were related to neurogenesis and neuronal differentiation, which led us to hypothesize that the defects in neuronal differentiation observed might not be specific to the dopaminergic system but the general neuronal population. Accordingly, we measured the levels of TUJ1+ and MAP2+ cells which are significantly lower at every time point assessed between DIV15 and DIV90 in the PD models (Supplementary Fig. 6a, b), thereby confirming a generalized neuronal deficit in GBA-N370S mutant MOs. These results were confirmed by western blot (WB) analysis of TUJ1 (Supplementary Fig. 6c).

In order to investigate whether the reduction in the neuronal population is already present during the early differentiation process, we evaluated the amount of neuroblast-like cells. These were identified by the expression of doublecortin (DCX), which is expressed during a limited phase in the brain development and is a reliable marker of neurogenesis^35,36^, and Nestin, a neural stem cell marker whose expression persists in immature neurons^37^. The proportion of DCX+/Nestin+ cells is significantly decreased in the PD models (Supplementary Fig. 6d, e), confirming that an immature neuronal population is not prevalent in GBA-N370S MOs and there is indeed an impairment in the differentiation process.

Finally, this neuronal differentiation defect was further confirmed by extracellular neuronal activity recordings using a multi-electrode array (MEA), which showed lower electrophysiological activity in patient-specific MOs (Figs. 4h and S6f, g).

To determine if the lower proportion of dopaminergic neurons in patient-specific MOs was simply a consequence of an impaired general neuronal differentiation, we quantified the amount of TH+ neurons and normalized to the total amount of neurons. This analysis confirmed that although general neurogenesis was impaired, the DA system was even more profoundly affected (Fig. 4i). Altogether, these data indicate that the amount of dopaminergic neurons is reduced in patient-derived midbrain organoids, hence recapitulating the major neuropathological feature of PD.

### Increased number of neural progenitor cells in GBA-N370S mutant MOs in cell cycle arrest

**Fig. 5:**
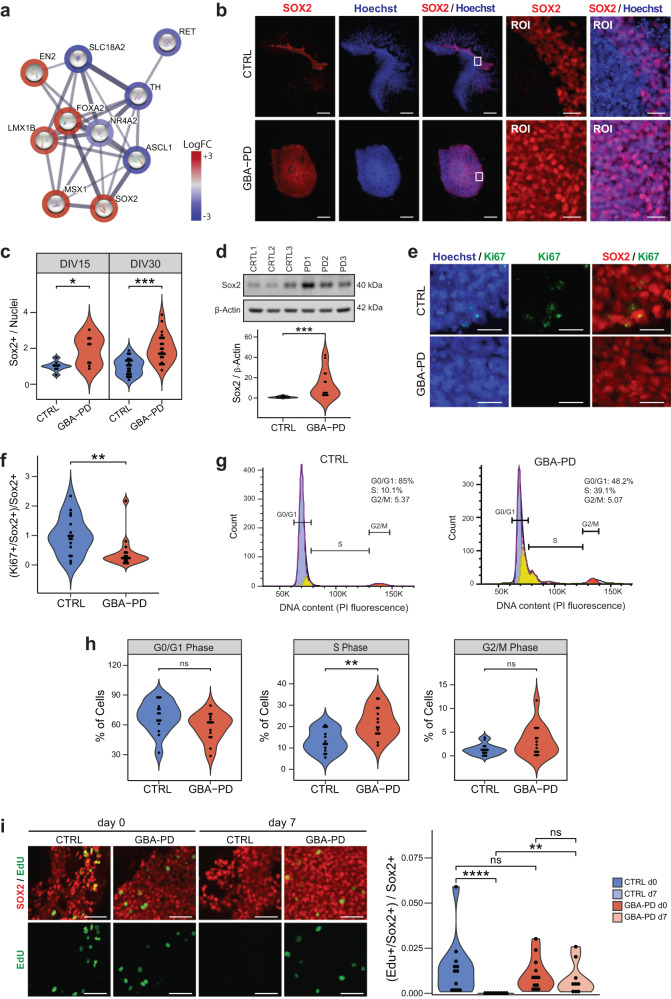
Increased levels of SOX2 and cell cycle arrest in GBA-PD organoids. **a** DEGs involved in dopaminergic differentiation (PathCards) depicted via protein–protein associations obtained from the STRING database. The border of the nodes represents the log fold-change (logFC) of the gene expression in the comparison of control GBA-PD organoids vs controls. Edges depict protein–protein associations. **b** Representative immunofluorescence staining of SOX2 (red) in midbrain organoid sections at DIV15 (CTRL1 and PD1), along with an amplified region of interest (ROI) of the original. Nuclei stained with Hoechst 33342 (blue), scale bar is 200 μm and 25 μm, respectively. **c** Quantitative analyses of SOX+ population shows increased proportion of cells expressing the neural stem cell marker in GBA-PD MOs at DIV15 and DIV30. Each data point represents the average of technical replicates for each independent differentiation. Values are normalized to the mean of the controls per experiment. Wilcoxon T-test; *p < 0.05, **p < 0.01, *n* = 3. **d** Validation of the immunofluorescence results by immunoblotting against SOX2 in whole cell lysates obtained from organoids at DIV15. The data represent a summary of four independent differentiations. Values are normalized to the mean of the controls per experiment. Wilcoxon T-test; ***p < 0.001. **e** Representative images CTRL2 and PD2 expressing SOX2 (red) and Ki67(green) at DIV15. Nuclei stained with Hoechst 33342 (blue) (scale bar, 20 μm). **f** Decreased proportion of proliferative neural stem cells in mutant MOs (DIV30) compared to controls, represented by cells expressing both SOX2 and Ki67. Values are normalized to the mean of the controls per experiment. Wilcoxon T-test; *p < 0.05. **g**, **h** Propidium iodide (PI) fluorescence profiles of CTRL3 and PD3 with cell cycle distribution (Watson pragmatic model) (G). Percentage of cells in each cycle phase analyzed by flow cytometry using propidium iodide showing accumulation of cells in the S-phase at DIV30 in GBA-PD organoids. The experiment was repeated five times using organoids from five independent differentiations. Wilcoxon T-test; *p < 0.05. **i** Representative images (left) of EdU staining (green) for evaluation of the proliferation of SOX+ neural precursors (red) at the day of the exposure (day 0) and 7 days after the initial exposure to EdU (day 7). Images correspond to organoids at DIV30 of CTRL1 and PD1 cell lines (scale bar, 50 μm). Respective quantification (right) of the proportion of neural precursors with a positive signal for EdU showing a significant loss of the EdU staining in CTRL organoids at day 7 after EdU exposure. Data represents a summary of at least three independent differentiation experiments. Kruskal–Wallis with post hoc Dunn tests; **p < 0.01, ****p < 0.0001.

To better understand the mechanisms leading to decreased amounts of DA neurons, we investigated the genes involved in dopaminergic differentiation (Fig. 5a), among the top 10 DEGs, a downregulation of NR4A (NURR1) was identified, which is a well-known essential transcription factor for the differentiation, maturation, and maintenance of midbrain DA neurons^38–40^. TH expression was equally decreased, consistent with our immunofluorescence and WB data. Additionally, RET receptor tyrosine kinase, was downregulated along with its interactive partners, including GDNF, a known neurotrophic factor (Supplementary Fig. 7a).

Interestingly, FOXA2 and SOX2 were upregulated (Fig. 5a), both of which are known to be highly expressed in dopaminergic neural progenitors^41,42^. An increase of FOXA2+ progenitor cells has been reported in MOs from PD patients carrying the LRRK2-G2019S mutation^15^. This might indicate that although the neural progenitors do not differentiate into neurons, they do not differentiate into other cell types either and remain in an undifferentiated state instead. Thus, we quantified the amount of cells expressing the neural progenitor marker SOX2 (Fig. 5b, c) and FOXA2 (Supplementary Fig. 7b–d), which proved to be significantly higher in GBA-PD organoids compared to controls. We confirmed these results via WB (Fig. 5d) and found SOX2 levels to remain markedly increased at later timepoints as well (Supplementary Fig. 7e). This would indicate a defect in the differentiation of neural stem cells, which probably leads to an enlarged progenitor pool in GBA-PD organoids.

We hypothesized that an upregulation of the neural progenitor population might be a compensatory response to an impaired DA neuron specification, as similar compensatory mechanisms have been reported in PD before^43^. However, such a response would be accompanied by an increase in organoid size and proliferation markers. Nevertheless, we did not find differences in size between the two conditions throughout the differentiation process (Supplementary Fig. 8a, b). Likewise, we measured the expression of the proliferation marker Ki67 at DIV15 and DIV30 and found no evidence of increased levels in patient-derived organoids (Supplementary Fig. 8c). To exclude that differences were masked by other cell types, we assessed the proliferating neural stem precursors by quantifying cells expressing both Ki67 and SOX2. Surprisingly, we found a decrease in the co-expression of the two markers in patient-specific MOs (Figs. 5e, f and S8d), implying that even though there is a higher proportion of neural progenitors in the GBA-PD condition, they have a lower proliferative capacity. This is an indicator that the progenitors in GBA-PD MOs might be under cell cycle arrest. In the pathway enrichment analysis of the transcriptomics data, we indeed detected several pathways related to cell cycle regulation (Supplementary Fig. 8e). Thus, we analyzed the cell cycle by flow cytometry using propidium iodide (PI) staining. Although we found no differences in the proportions of cells in G0–G1 or G2–M phase, there was a significant accumulation of cells in the S-phase population (Fig. 5g, h). To further monitor S-phase progression, the thymidine analog, EdU (5-ethynyl-2′-deoxyuridine) was used to tag cells in S-phase as it is incorporated into cellular DNA during replication. As expected, immediately after the EdU pulse, the proportion of neural precursors that incorporated the dye was similar between CTRL and GBA-PD DIV30 MOs (Figs. 5i and S9). However, because of dilution through multiple cell divisions, the EdU signal was lost completely in pulse-labeled progenitors of control organoids after 7 days from initial EdU pulse, whereas it was retained in GBA-PD organoids (Figs. 5i and S10), indicating that the neural precursors of patient-derived MOs did not divide and were therefore arrested in S-phase.

### Neural progenitors of GBA-PD organoids display signs of oxidative damage and cellular senescence

**Fig. 6:**
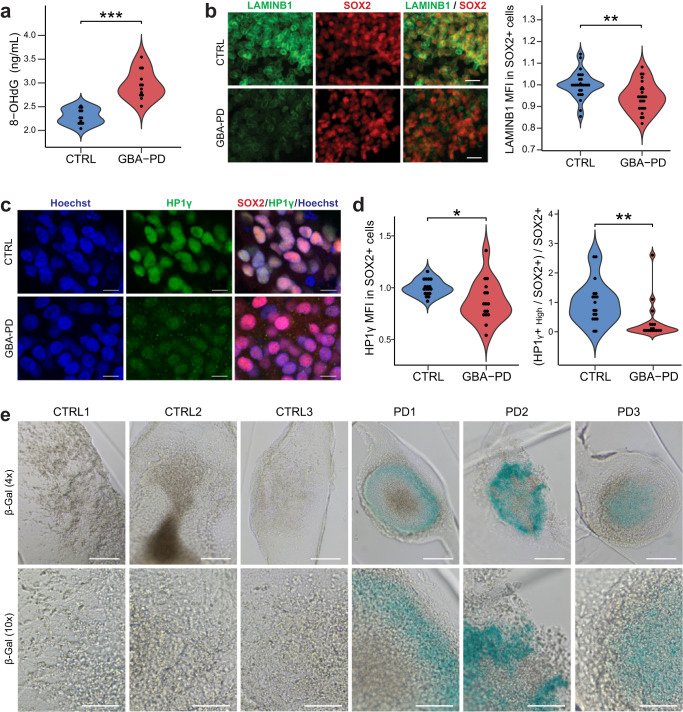
DNA oxidative damage and signs of senescence in GBA-PD organoids. **a** Extracellular concentration of 8-OHdG in DIV30 organoids measured by ELISA. The data represent a summary of four independent differentiation experiments for all cell lines. Wilcoxon T-test; ***p < 0.001. **b** High content automated image analysis showed decreased MFI of LAMINB1 (green) within Sox+ population (red). Data is a summary of eight independent differentiation experiments, normalized to the average of controls per organoid batch. Wilcoxon T-test; **p < 0.01 (scale bar, 20 μm). **c**, **d** Representative images of HP1γ (green) and SOX2 (red) in CTRL2 and PD2 organoid sections acquired at 60× (C) (scale bar, 10 μm). Quantification of MFI of HP1γ within the SOX2+ population (D left) and proportion of neural precursor cells (SOX2+) expressing high levels of HP1γ (D right) in DIV30 organoid sections acquired at 20× using the automated image analysis pipeline. Data represents a summary of five independent differentiation experiments normalized to the mean of the controls per batch. Wilcoxon T-test; *p < 0.05, **p < 0.01. **e** Senescence-associated β-galactosidase staining (blue) of DIV30 organoids (scale bar, 200 μm, 4×; 100 μm, 10×).

A common trigger of cell cycle checkpoint activation is DNA damage^44^, and a significant amount of the damage is caused by reactive oxygen species (ROS)^45^. Moreover, oxidative stress is known to play an important role in PD pathogenesis, therefore we assessed DNA oxidative damage by measuring the levels of 8-Hydroxydeoxyguanosine (8-OHdG), a major oxidative DNA-damage product^45^. The levels of 8-OHdG released into the medium was significantly increased in the GBA-N370S condition (Fig. 6a), indicating that patient-derived MOs are under severe oxidative stress, thereby restraining the cell cycle progression.

Persistent DNA damage response may result in a permanent cell cycle arrest, leading to cellular senescence^46,47^. A well-established marker for senescence is the loss of nuclear LAMINB1^48^. Accordingly, we quantified the expression of nuclear LAMINB1 in the neural precursor population and were able to detect a significant decrease (Figs. 6b and S11). The senescent phenotype is associated with the loss of heterochromatin, which can be assessed by immunostaining of heterochromatin protein 1 gamma (HP1γ)^49,50^. Organoids from GBA-PD patients present a lower mean fluorescence intensity (MFI) than control-derived organoids (Figs. 6c, d left and S12a), despite the presence of a bright dotted pattern in GBA-PD MOs resembling senescence-associated heterochromatin foci (SAHF)^51^, which is absent in control MOs (Supplementary Fig. 12b, c). When the threshold of the image analysis quantification was adjusted to account for this, in order to identify a population that uniformly expresses high levels of HP1γ, the decrease in GBA-PD MOs became more significant (Fig. 6d right). Moreover, the activity of the lysosomal enzyme beta-galactosidase (β-gal) is commonly used as a marker for senescent cells^52^ and increased activity of this enzyme in GBA-PD MOs was already shown in (Supplementary Fig. 3b). To confirm these results, we performed senescence-associated beta-galactosidase (SA-β-gal) staining, showing an accumulation of SA-β-gal positive cells in GBA-PD organoids (Fig. 6e).

Collectively, these data suggest that GBA-N370S mutation causes cell cycle arrest and cellular senescence in neural progenitor cells, resulting in impaired neurogenesis.

## Discussion

In this study in silico metabolic network modeling identified several pathways that potentially play a role in GBA-N370S-associated PD. These pathways could not only be validated by extensive phenotyping but also led to the discovery of potential mechanisms of action of GBA-N370S on neurodevelopment and in PD pathogenicity.

By using patient-specific midbrain organoids we confirmed decreased GCase activity, a dysregulation of autophagy, altered mitochondrial function and highlighted the role of the heterozygous GBA-N370S mutation in lipid dysregulation. We showed differences in steady-state levels of several lipid classes as well as differences in lipid composition within the same class, which may decisively distort cell homeostasis. For instance, we observed a decrease in triglycerides (TG), which could have an effect in α-synuclein aggregation in later stages of the disease, as TGs are protective against α-synuclein cytotoxicity and ER trafficking defects^53,54^. Likewise, the depletion of PE, might explain the lower levels of LC3-II observed, as in order for LC3-I to become LC3-II upon autophagy induction, it needs to be conjugated to PE^55,56^. Interestingly, a decrease in PE has also been observed in PD patients using magnetic resonance spectroscopic imaging^57^.

While homozygous mutations in the GBA gene are associated with accumulation of GlcCer in Gaucher disease, it remains controversial whether heterozygosity is equally associated with substrate accumulation as it has not yet been demonstrated in GBA-PD patient brains^28,58^. In patient-derived iPSC neuronal cultures, both an accumulation^59^ and unchanged levels^18^ have been reported. Consistent with the latter, we did not observe an increase in total levels of hexosylceramides. However, it is of note that differences in GlcCer could have been masked by the levels of galactosylceramide, since technically it was impossible to distinguish the two.

Moreover, we demonstrated that the impaired dopamine transport stems from a lower number and notably more dysfunctional DA neurons. A defective specification of DA neurons will likely affect their vulnerability. The transcription factor NURR1, essential for the differentiation, maturation, and maintenance of mDA neurons^60^ was significantly decreased in GBA-PD organoids. An early reduced expression of NURR1 has been shown to result in a lower DA yield and an increased susceptibility of these neurons^61,62^. Moreover, NURR1 is essential for the expression of the RET protein in midbrain DA neurons^63,64^, consistently, the Ret-GDNF signaling pathway was downregulated in GBA-PD organoids. GDNF is an important neurotrophic factor in the midbrain DA system and increasing evidence suggests that GDNF/Ret signaling is an important modulator of development and maintenance in midbrain dopaminergic neurons^65,66^. Together, these results suggest that neurodevelopment is strongly impaired in GBA-N370S midbrain organoids.

The reduction of neurons during differentiation was associated with an accumulation of undifferentiated cells. This observation offers an explanation for the big differences in flux variability analysis (FVA) for the glycolysis pathway between patients and controls when maximizing for ATP production, since neural stem cells depend mainly on glycolysis for meeting their energetic demand^67^. These neural stem cells were, however, not proliferative, which was accompanied by increased DNA oxidative damage, leading to cell cycle arrest at the S-phase, thereby potentially explaining the lower amount of differentiated neurons in GBA-PD organoids. Moreover, these non-proliferative neural stem cells showed signs of premature senescence, supported by a loss of LAMINB1, as well as an upregulation of β-gal and a downregulation of HPIγ, three well-known hallmarks of senescence^49,50,52^. But although there was a general decrease of HPIγ expression, GBA-N370S organoids exhibited a punctate staining, as opposed to control organoids, that might indicate the presence of senescence-associated heterochromatin foci (SAHF), which are thought to contribute to the maintenance of the senescent phenotype^51^.

An altered transition between a neural precursor state and a differentiated neuron has already been reported in other forms of PD^15,68^. Nevertheless, the neurodevelopmental contribution of GBA mutations has not been extensively investigated. A previous study showed that iPSC-derived neurons from monozygotic twins harboring the heterozygous GBA-N370S but clinically discordant for PD had reduced capacity to synthesize and release dopamine^69^, although differentiation efficiency was not assessed. Moreover, in a Gaucher disease in vitro model harboring a homozygous mutation in the GBA gene, the ability of neural progenitors to differentiate to DA neurons was significantly reduced^70^. However, impaired DA neurogenesis has not yet been described in GBA-PD in vitro models, this could be explained by the differences in DA neuron differentiation protocols or the number of cell lines used. This further proves that iPSC-derived 3D organoids, which have a higher resemblance to in vivo cell organization and organ structure than standard 2D cultures^13,71^, can be used to identify potential early developmental defects in neurodegenerative diseases such as PD.

β-Glucocerebrosidase (GCase) activity is reduced not only in mutation carriers but also in idiopathic PD and healthy individuals at older age^72,73^, pointing toward a general role for GCase in neurodegenerative processes. It would be necessary to find out from a mechanistic point of view, if autophagy upregulation would be enough to increase the neuronal differentiation efficiency as observed in other organoid PD models^68^, or whether the phenotypes observed are a consequence of other pathological processes such as ER stress or oxidative damage, which are more likely to cause cell cycle arrest and the subsequent low differentiation yield. On the other hand, emerging evidence supporting a role of lipid metabolism in neural stem cell proliferation and neurogenesis has gained attention recently^74,75^. In line with this, many phosphoinositide signaling genes were differentially expressed in GBA-PD MOs, some of which are known to be required for neural tube morphogenesis and differentiation during embryonic development, such as PTEN, GAP43, SYT1, and CFL1^33,76^. This offers exciting possible mechanisms through which an altered lipidome due to mutant GCase affects stem cell regulation and neuronal differentiation. Pharmacological rescue of GCase activity using recombinant GCase or chemical chaperones will allow further exploration of the mechanisms by which GBA-N370S mutation leads to the developmental defects observed and may provide promising therapeutic strategies to treat PD.

In summary, we here show that PD patient-specific midbrain organoids recapitulate cardinal disease features in vitro. Furthermore, we used this model to identify a cellular process, stress-induced cell cycle arrest of neural progenitor cells, as potentially underlying cause for deficiencies in dopaminergic neurons. These findings support the notion that PD has a neurodevelopmental component and opens unexplored avenues for therapeutics developments targeting these mechanisms.

## Methods

### iPSCs, mfNPCs, and organoid culture

Patient GBA-PD1 sample was obtained from Coriell Institute and Patients GBA-PD2 and GBA-PD3 were provided by University College London. Healthy controls 1 and 2 were generated at the Max Planck Institute and healthy control 3 was provided by StemBANCC. From each donor, one clone per iPSC line was used in this study. The use of existing iPSC lines obtained from previous studies was approved by the local ethical committee (Comité National d’Ethique de Recherche), the work with these cells is in compliance with the national guidelines. More information is detailed in (Supplementary Table 1). Full characterization of iPSCs is available at 10.17881/xfh3-a153. iPSCs were cultured in Essential 8 medium (Thermo Fisher, A1517001) with 1% Penicillin/Streptomycin (Invitrogen, 15140122) in Matrigel-coated plates (Corning, 354277). Splitting was performed using EDTA 0.5 mM (Invitrogen, 15575020) and ROCK inhibitor Y-27632 (Merck Milipore, cat no. 688000) was added to the media at 10 μM for 6–24 h after seeding.

Midbrain floor plate neural progenitor cells (mfNPCs) were derived from iPSCs as previously described in ref. ^15^. mfNPCs were maintained in Matrigel-coated plates and cultured in freshly supplemented N2B27 as described in ref. ^16^. In order to obtain a homogeneous population of mfNPCs, the cells were passaged at least 5 times before doing an immunofluorescence staining to confirm the neural stem cell identity, which we validated by the expression of the neural stem cell markers SOX2 and NESTIN and we confirmed absence of expression of PAX6, thereby excluding a dorsal identity (Supplementary Fig. 1).

Once the quality of the derivation was confirmed by immunostaining, ventral midbrain organoids were generated as detailed in ref. ^16^, using a reduced seeding density of 6000 cells. For each organoid generation, the passage of mfNPCs was the same between controls and GBA-PD cell lines. The MOs were embedded in Geltrex (Invitrogen, cat no. A1413302) at day 8 of differentiation and kept at 37 °C, 5% CO_2_ under dynamic conditions (80 rpm) for up to 90 days or were left in 96-well ultralow adhesion plates (round bottom, Corning).

### GBA-N370S mutation screening

Cell lines coming from GBA-PD patients were screened for the N370S mutation in the GBA gene by extracting genomic DNA from blood samples using the GenElute™ Blood Genomic DNA Kit (Sigma, NA2020-1KT), PCR reactions were carried out using GoTaq® G2 Hot Start Master Mix (M7423, Promega). Primer sequences were F: TGTGTGCAAGGTCCAGGATCAG, R: ACCACCTAGAGGGGAAAGTG. Samples were sent for sequencing to Microsynth Seqlab and mutations were confirmed (see “Resource availability”).

### Cell cycle analysis by flow cytometry

For flow cytometry analysis, four embedded organoids of each cell line were pooled and incubated with 500 μL of papain (0.18% Papain, Sigma; 0.04% EDTA, Sigma; 0.04% L-Cystein; DMEM-F12, Invitrogen) for 40 min at 37 °C. After 40 min, papain was removed and 300 μL of Accutase (Sigma) were added, organoids were then immediately disrupted into a homogeneous suspension by pipetting, in order to stop the digestion, 1 mL of PBS containing 0.5% trypsin inhibitor and 0.5% of BSA was added. The samples were then centrifuged for 5 mins at 500 × *g* and washed once in ice-cold PBS. For cell fixation, 70% (v/v) of ice-cold ethanol was added to the pellet dropwise while vortexing. Cells were fixed for 30 min at 4 °C on a rotor. After fixation, cells were washed twice with 1 mL of PBS and centrifuged at 850 × *g* for 5 min.

The pellet was then resuspended in 250 μL of propidium iodide working solution (PI; P4864, Sigma 40 μg/mL, and RNase A 20 μg/mL in PBS) and incubated for 15 min at room temperature.

Cells were then analyzed by fluorescence-activated cell sorting (FACS) on the BD LSRFortessa Cell Analyzer using the 488 nm laser for excitation. Data were analyzed using FlowJo v10 software and cell cycle phases were quantified by the Watson Pragmatic algorithm.

### GCase activity

GCase activity was measured as described previously^18,77^. Briefly, non-embedded organoids were lysed in GCase lysis buffer (citrate phosphate buffer supplemented with 0.25% (v/v) Triton X-100 and 0.25% (w/v) taurocholic acid pH 5.4). Following incubation on ice for 30 min, samples were centrifuged for 20 min at 4 °C, 20,000 × *g*. The supernatant was incubated with 1 mM 4-methylumbelliferyl β-D-glucopyranoside (Sigma, M3633) and incubated at 37 °C for 40 min. The reaction was stopped by adding equi-volume of glycine (1 M, pH 12.5) and resulting fluorescence detected on a Microplate Reader Cytation5M (BioTek). Excitation at 355 nm and emission 460 nm was used. GCase activity was normalized to the total protein content of the lysates using the Pierce™ BCA Protein Assay Kit (Thermo Fisher). Values from samples treated with 3 mM conduritol B epoxide (Santa Cruz, sc-201356) were subtracted from non-CBE-treated samples to obtain activity derived from GBA1. GCase activity was normalized to the mean of the controls per experiment and results were expressed as percentage of controls.

### Other lysosomal enzyme assays

Measurements of total β-hexosaminidase and β-galactosidase enzymatic activity were determined in as described previously^78^. 30DIV non-embedded organoids were lysed in 80 μL of citrate-phosphate buffer (10 mM, pH 4.2) containing 0.5% BSA 0.5% and Triton X-100. 25 μL of either 4-methylumbelliferyl-N-acetyl-b-D-glucosaminide (MUG, 0.05 mM; Sigma, M2133) or 4-methylumbel-liferyl-β-D-galactopyranoside (MUbGal, 0.56 mM; Sigma, M1633) were added to 10 μL of organoid lysate as substrate and incubated at 37 °C for 1 h. The reaction was stopped by the addition of 200 μl of 0.1 M 2-amino-2-methyl-1-propanol (pH 10.5) and fluorescence was measured (excitation 365 nm; emission 450 nm) using the Microplate Reader Cytation5M (BioTek). The readouts were not normalized to the protein content due to the interference of BSA from the extraction buffer with the protein quantification. To measure β-galactocerebrosidase activity, samples were processed as described before^79^. Briefly, 30DIV non-embedded organoids were lysed in 0.25% Triton X-100 and 0.25% sodium taurocholate, 10 μL of the lysate were then added to 20 μL of substrate reagent consisting of 0.4 mM 6-hexadecanoylamino-4-methylumbelliferyl-β-D-galactopyranoside (HMUGal; Creative Enzymes, CSUB-0720), 0.3% sodium taurocholate, and 0.18% oleic acid in citric acid/phosphate buffer (pH 5.2). The enzymatic reaction was carried out at 37 °C for 6 h, and then was stopped by addition of 60 μL 0.2 M glycine-NaOH buffer (pH 10.7) with 0.1% SDS and 0.15% Triton X-100. Measured fluorescence (excitation 404 nm; emission 460 nm) was normalized to total protein concentration (Pierce™ BCA Protein Assay Kit. Thermo Fisher).

### Dopamine extracellular release

Dopamine ELISA (Immusmol BA-E-5300) was performed for the quantitative determination of dopamine secreted by midbrain organoids at DIV60 from five independent non-embedded organoid differentiations. Media was changed 2 days before collection, on the day of the collection, 270 µl of medium were taken and diluted with 30 µl of HCl buffer (0.01 N HCl, 4 mM Na2O5S2, and 1 mM EDTA), they were then snap-frozen in liquid nitrogen and stored at −80 °C until the day of the analysis. The ELISA was performed according to the manufacturer’s instructions with 50 µl sample volume. Values were normalized to the size (area) of the organoids.

### Western blot

For western blot, five non-embedded organoids were lysed using RIPA buffer (Abcam) with Complete protease inhibitor cocktail (Roche) for 20 min on ice. In order disrupt DNA, lysates were sonicated for 10 cycles (30 s on/30 s off) using the Bioruptor Pico (Diagenode). Samples were then centrifuged at 4 °C for 20 min at 14,000 × *g*. The protein concentration was determined using the Pierce™ BCA Protein Assay Kit (Thermo Fisher). Samples were brought to the same concentration and boiled at 95 °C for 5 min in denaturating loading buffer. All samples derived from the same organoid batch were loaded on the same gel and for each figure panel, all blots were processed in parallel and derive from the same experiments. Protein separation was achieved using SDS polyacrylamide gel electrophoresis (Bolt™ 4–12% Bis-Tris Plus Gel, Thermo Fisher) and transferred onto PVDF membrane using iBlot™ 2 Gel Transfer Device (Thermo Fisher). Membranes were blocked for 1 h at RT in 5% skimmed milk powder in PBS before incubating overnight at 4 °C with the primary antibodies prepared in 5% BSA and 0.02% Tween (see Supplementary Table 2 for list of antibodies used). Membranes were then washed 3× for 5 min with PBST and incubated with DyLight™ secondary antibodies (anti-rabbit IgG (H + L) 800 or anti-mouse IgG (H + L) 680). Membranes were scanned in the Odyssey® Fc 2800 Imaging System. Western blots were analyzed using ImageJ software. All uncropped western blots are shown in Supplementary Fig. 13.

### Immunofluorescence stainings for cell characterization

Immunofluorescent stainings for iPSCs and mfNPCs were performed as described in ref. ^80^ and ref. ^15^, respectively. Cells were fixed with 4% paraformaldehyde for 15 min at RT, then washed 3× with PBS for 5 min at RT and permeabilized using 0.5% Triton X-100 in PBS for 15 min at RT. Blocking was performed for 1 h at RT with 10% FCS in PBS. Incubation with the primary antibodies (see Supplementary Table 2) was done overnight at 4 °C in antibody buffer containing 0.3% Triton X-100 and 10% FCS in PBS. Cells were then rinsed three times in PBS for 5 min and incubated with fluorochrome-conjugated secondary antibodies diluted in antibody buffer for 1 h and protected from light. Nuclei were counterstained by Hoechst 33342 (Life Technologies, cat no. H21492). Cells were washed three times with PBS and mounted with Fluoromount-G mounting. Imaging was done with a confocal microscope (Zeiss, LSM 710). A commercially available control cell line (Alstem, Cat# iPS15) was derived into small molecule neural precursor (smNPC)^81^ and used as a positive control for Pax6 immunostaining (Supplementary Fig. 1b).

### Immunofluorescence stainings of organoid sections

Immunofluorescence staining of organoids was carried out using a modified published protocol^16^. Embedded organoids were fixed with 4% paraformaldehyde overnight at RT and washed 3× with PBS for 15 min. One organoid per cell line, condition and experiment were embedded in 3% low-melting point agarose, the solid agarose block with the organoid was sectioned into 70 µm sections using a vibrating blade microtome (Leica VT1000s). The sections were permeabilized and blocked with blocking buffer (BB) (2.5% donkey serum or normal goat serum, 2.5% BSA, and 0.1% sodium azide) containing 0.5% Triton X-100. Sections were incubated for 72 h at 4 °C on an orbital shaker with primary antibodies (see Supplementary Table 2) in BB containing 0.1% Triton X-100.

Incubation with secondary antibodies and mounting of the sections were performed as described in ref. ^16^.

For high-content image analysis, stained sections from at least three independent organoid differentiations were acquired on Yokogawa CV8000 high content screening microscope with a ×20 Objective (33 planes, 3,2 μm interval).

For quantitative analysis at ×60, a confocal laser scanning microscope (Zeiss LSM 710) was used, two randomly selected fields per organoid section were acquired of three independent organoid differentiations, keeping the same acquisition settings.

For qualitative images, organoid sections were acquired with a confocal laser scanning microscope (Zeiss LSM 710) with either a ×20, ×40, or ×60 objective. Three-dimensional surface reconstructions of confocal z-stacks were created using Imaris software (Bitplane).

Representative images were cropped, rotated, or rescaled using Adobe illustrator for visualization purposes, all originals can be found at 10.17881/xfh3-a153.

### EdU incorporation assay

For EdU incorporation assay, 30DIV embedded organoids were incubated with Edu for 8 h and then fixed with 4% PFA immediately or maintained in culture for 7 days after EdU removal. Staining was performed using the Click‐iT Plus EdU Alexa Fluor Imaging Kit (Thermo Fisher, Cat # C10638) according to the manufacturer’s protocol, sections were then immunostained for additional markers using the protocol described above.

### Image processing and analysis

Immunofluorescence 3D images of each organoid were processed and analyzed in Matlab (2020a, Mathworks) using a custom image-analysis algorithm as described previously^82,83^.

### Transcriptomics analysis

Total RNA was isolated from 3 independent organoid generations at DIV30, for each sample, 8 embedded organoids of the same batch were pooled from CTRL1, CTRL2, PD1, and PD2 using Trizol reagent (cat. no. 15596026, ThermoFisher) according to the manufacturer’s protocol. mRNA library preparation and sequencing were conducted as described before^84^. Sequencing of samples was done in two individual runs, PD1 and CTRL2 were analyzed in parallel during the first run, CTRL2 and PD1 were analyzed together during the second run.

### Analysis of transcriptomics data

Gene expression analysis based on the RNAseq data was done following the method described in ref. ^84^. *p*-value significance scores from the two individual datasets (PD1 vs CTRL2 and PD2 vs CTRL1) were combined using the weighted meta-analysis method by Marot and Mayer^85^, focusing only on the genes with shared sign of the log fold-change. The final integrated p-values were then adjusted for multiple hypothesis testing using the Benjamini–Hochberg approach. The combined log fold change is expressed as the mean of the log fold change from the two datasets.

### Lipidomics analysis

For lipidomics analysis, four embedded organoids at DIV30 were pooled per cell line for each experiment. Analysis was performed on two of the controls (CTRL1 and CTRL2) and all of the GBA-PD lines (PD1 and PD2 and PD3). On the day of the collection, organoids were washed once with ice-cold PBS and snap-frozen with liquid nitrogen. Lipid species were analyzed by Lipometrix (http://www.lipometrix.be) using HILIC LC-MS/MS. Results were normalized to DNA content. Analysis was carried out on samples from four independent organoid generations. *p*-values were calculated using a one-way ANOVA test, assuming equal variance (homoscedasticity) in the 2 groups. FDR adjusted *p*-values were calculated using the Benjamini–Hochberg^86^ procedure.

### Microelectrode array (MEA)

MEA recordings were conducted using the Maestro system from Axion BioSystems using a protocol adapted from ref. ^84^. Ten-day-old non-embedded organoids were placed on a 96-well MEA plate containing 8-electrodes per well and precoated with 0.1 mg/ml poly-D-lysine hydrobromide (Sigma, P7886) and 10 µg/ml laminin (Sigma, L2020), a droplet of geltrex (Invitrogen, A1413302) was added on top to secure the organoid on the right position. The recording of the firing activity was carried out as described before^13,84^. The Axion Integrated Studio software (AxIS 2.1), was used to analyze MEA recordings. To minimize false positives and missed detections, a Butterworth band pass filter with 200–3000 Hz cutoff frequency and a threshold of 6 x SD were used. Electrodes were defined as active when the average activity detected was ≥5 spikes/min. Neural stat compiler files generated from the recordings were used for the data analysis. Further details regarding the analysis were previously described^84^. Script for data analysis is uploaded in the corresponding Gitlab repository.

### Metabolic modeling

RNA sequencing Transcripts Per Kilobase Million (TPM) reads were filtered to exclude genes with expression of 0 among all technical replicates of each cell line. After filtering, the median of TPM expression between technical replicates was calculated. In case of gene isoforms, the isoform with the highest median value was considered. Using clusterProfiler package (version 3.9) in R (version 3.6.2) gene symbols were assigned to EntrezIDs, which are required for the model generation.

For every cell line (two mutants and two healthy controls) a context-specific metabolic model was reconstructed with the rFASTCORMICS pipeline^25^ using Recon3^24^. Models were constrained by the media composition. Generated models were further analyzed using the COBRA toolbox^87^ in Matlab (version 2019b).

Models were compared regarding their gene, reaction and metabolite composition. Shared genes, reactions, and metabolites between both control models (CTRL1 and CTRL2) and then between both GBA-PD models (PD1 and PD2) were identified using the intersect function of Matlab. Unique genes, reactions and metabolites for control models and mutant models were found using the setdiff function of Matlab. Identified reactions being uniquely present in healthy or PD models were assigned to Recon3 subsystems. Unique gene functional classification was analyzed using the David enrichment tool freely available online at https://david.ncifcrf.gov/home.jsp.

Flux variability analysis (FVA) for the subsystems of interest was done with model optimization for ATP demand using the IBM_CPLEX solver (version 12.10). Subsystems of interest were defined based on the sum of reactions being present uniquely in healthy or in PD models. For most of the subsystems this reaction count was directly correlating with the size of subsystem in Recon3, therefore the number of reactions was expressed as percentage of the total amount of reactions in the respective subsystem in Recon3. Subsystems with a total size larger than 20 reactions in Recon3 and with more than 20% of reactions being different between control and PD models were additionally considered for the FVA. Peptide metabolism and Keratan sulfate synthesis subsystems were not analyzed with FVA because they were not present in the control models, as no comparison would be possible. In addition, central energy metabolism subsystems—Glycolysis, Pentose Phosphate pathway, Citric acid cycle, and Oxidative phosphorylation were included in the downstream analysis.

A similarity index (SI) was calculated to assess the differences in flux variability for the subsystems of interest between healthy and PD models, using the formula:

$${\rm{SI}}=\max (0,(\min ({\rm{v}}1{\rm{max}} ,{\rm{v}}2{\rm{max}})-\max ({\rm{v}}1{\rm{min}},{\rm{v}}2{\rm{min}})+{\rm{eps}})/(\max ({\rm{v}}1{\rm{max}} ,{\rm{v}}2{\rm{max}})\min ({\rm{v}}1{\rm{min}},{\rm{v}}2{\rm{min}})+{\rm{eps}}))$$

Where v1max is the maximal predicted flux of model 1, v2max is the maximal predicted flux of model 2, v1min is the minimal predicted flux of model 1, v2min is the minimal predicted flux of model 2, and eps = 0 because SI was calculated only for the shared reactions between the models.

Since SI can be calculated between two models, we computed SI for the following comparisons: CTRL1vsPD1; CTR1vsPD2; CTRL2vsPD1; CTRL2vsPD2; CTRL1vsCTRL2 and PD1vsPD2. Then the mean value of SI of the comparisons CTRL1vsPD1; CTR1vsPD2; CTRL2vsPD1; CTRL2vsPD2 was further estimated to obtain a representative SI between CTRL vs GBA-PD models. SI of 0 represents a complete mismatch in flux variability between the models, and SI of 1 represents the highest similarity in flux variability.

### Extracellular flux analysis (SeaHorse measurements)

Non-embedded organoids were seeded in a XF 96-well spheroid plate (Agilent Technologies, 102905-100) coated with Cell-Tak (Corning, 354240) and analyzed using an XFe96 Extracellular Flux Analyzer (Seahorse Biosciences) at DIV30. Seahorse XF Cell Mito Stress Tests were carried out using sequential injections of Oligomycin (5 µM), FCCP (1 µM), and Rotenone plus Antimycin A (1 µM). Values were normalized to the size (area) of the organoids. Three baseline measures and three measurements after each compound injection were performed.

### DNA oxidative damage

8-hydroxy-2-deoxy Guanosine (8-OHdG) was measured in the extracellular medium of non-embedded organoids at DIV30 (Abcam, ab201734). The ELISA was performed following the manufacturer’s instructions.

### SA-β-Galactosidase staining

Staining was performed on PFA-fixed embedded organoid sections with the Senescence Detection Kit (Abcam, ab65351) following the manufacturer’s instructions. Images were acquired at 4× and 10× on an Olympus IX83 microscope and enhanced using ImageJ software to account solely for differences in the background levels of light.

### Statistical analysis

The data was processed in R software (R version 3.5.1 -- “Feather Spray”). First, data for each cell line and experiment was grouped by differentiation experiment and condition and the mean of the measurements was calculated. Statistical significance was tested using the Wilcoxon signed-rank test for comparison between the two conditions (Control and GBA-PD). The statistical analysis of EdU staining was done by comparing the groups with Kruskal–Wallis H test, followed by a post hoc Dunn test. Each data point in the graphs corresponds to the data from one cell line for one individual differentiation experiment.

For analysis of lipidomics data, *p*-values were calculated using a one-way ANOVA test, assuming equal variance (homoscedasticity) in the 2 groups (CTRL and GBA-PD). FDR adjusted *p*-values were calculated using the Benjamini–Hochberg procedure approach^86^. In order to compare the levels of all lipid species within the same lipid class, the sum of all species for each class was calculated and a Mann–Whitney U-test was used using GraphPad prism 9 to test the significance between the two conditions.

For gene-level differential expression analysis of transcriptomics data, *p*-value significance scores from the two individual datasets (PD1 vs CTRL2 and PD2 vs CTRL1) were integrated using the Marot and Mayer method^85^. The final combined *p*-values were then adjusted for multiple hypothesis testing using the Benjamini–Hochberg procedure.

### Reporting summary

Further information on research design is available in the Nature Research Reporting Summary linked to this article.

### Supplementary information

Supplementary Figures

Reporting summary

## Data Availability

All original and processed data as well as scripts that support the findings of this study are public available at this 10.17881/xfh3-a153. Gene expression datasets can be accessed on the Gene Expression Omnibus database under the identifier GSE208784.

## Code availability

All scripts used to obtain, analyze and plot the data are available at https://gitlab.lcsb.uni.lu/dvb/rosety_2022.
