# Supplementary material for: Impaired neuron differentiation in GBA-associated Parkinson’s disease is linked to cell cycle defects in organoids
Source: NPJ Parkinsons Dis. 2023 Dec 18;9:166. doi: 10.1038/s41531-023-00616-8 (PMC10728202; doi:10.1038/s41531-023-00616-8)
Supplement: Supplementary file 1 — Supplementary Figures [file 41531_2023_616_MOESM1_ESM.pdf]

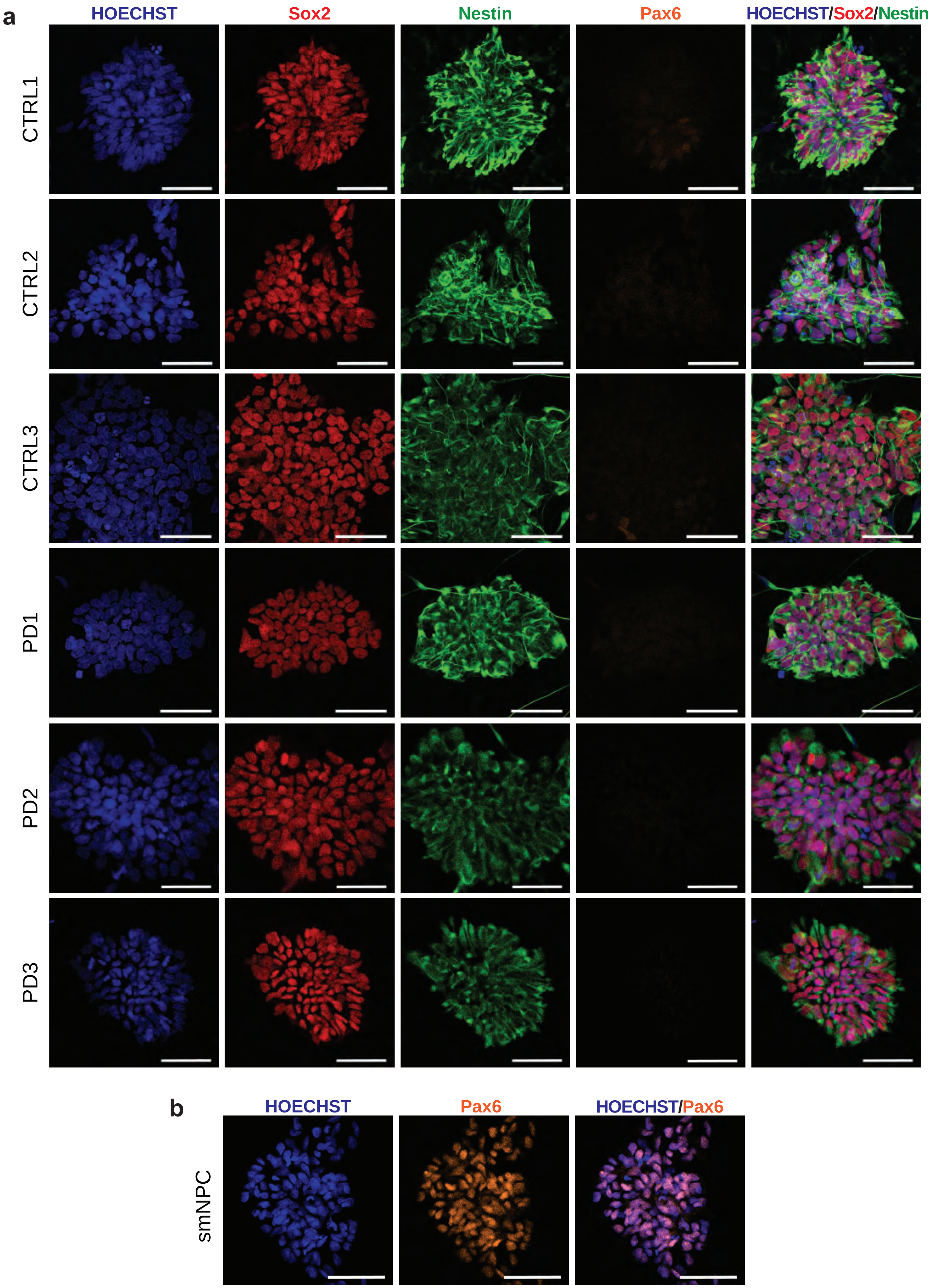
a
HOECHST
Sox2
Nestin
Pax6
HOECHST/Sox2/Nestin
CTRL1
CTRL2
CTRL3
PD1
PD2
PD3
b
HOECHST
Pax6
HOECHST/Pax6
smNPC

Supplementary Fig. 1. Derivation of mfNPCs from iPSCs.

a) Immunostaining of mfNPCs for Nestin (green), Sox2 (red) and Pax6 (orange). Nuclei were counterstained with Hoechst (blue) (scalebar 50 µm).
b) Positive control for Pax6 immunofluorescence staining of a healthy smNPC line Pax6 (orange). Nuclei were counterstained with Hoechst (blue) (scalebar 50 µm).

Supplementary Fig. 2

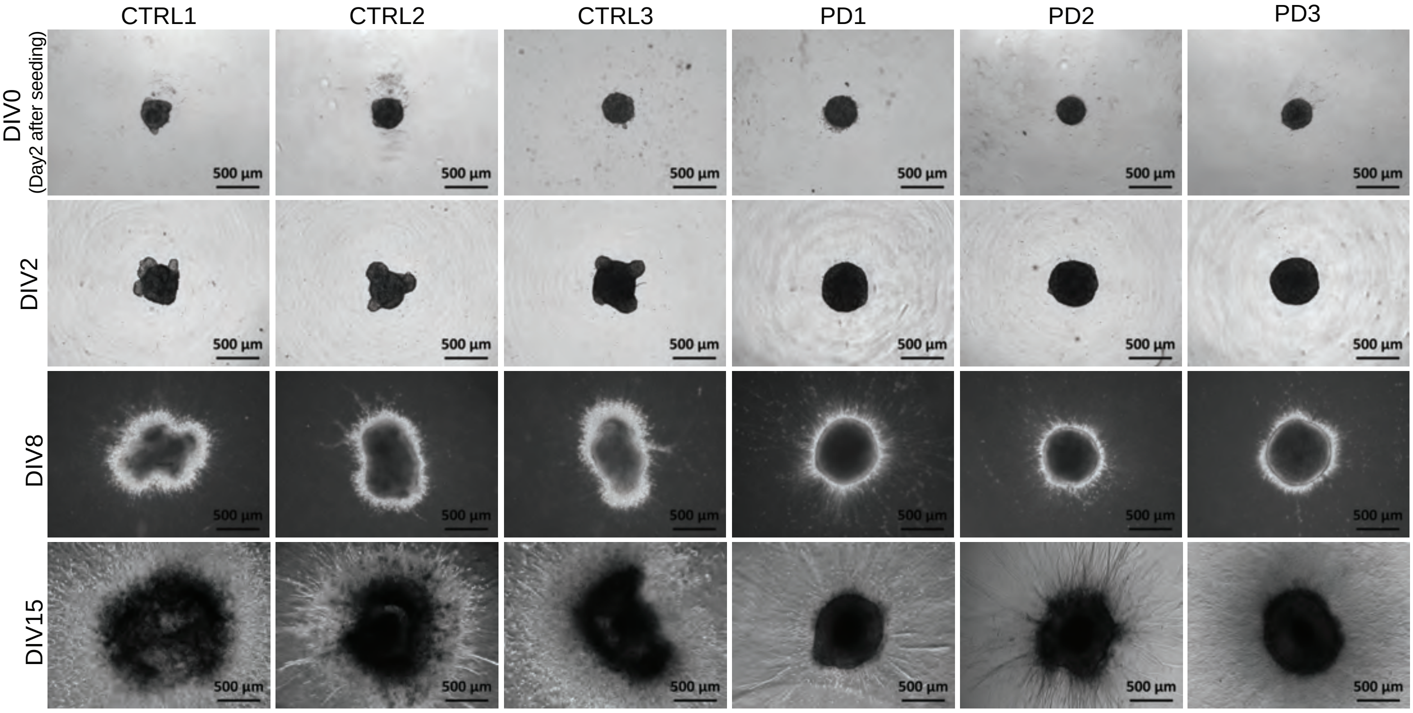

Supplementary Fig. 2. Brightfield images of organoid development.

Representative bright-field images showing organoid development from day 2 of the seeding (DIV0) to DIV15. Images from DIV0 and DIV2 correspond to non-embedded organoids, images from DIV8 and DIV15 correspond to organoids after embedding on DIV6 (scalebar 500 µm).

Supplementary Fig. 3

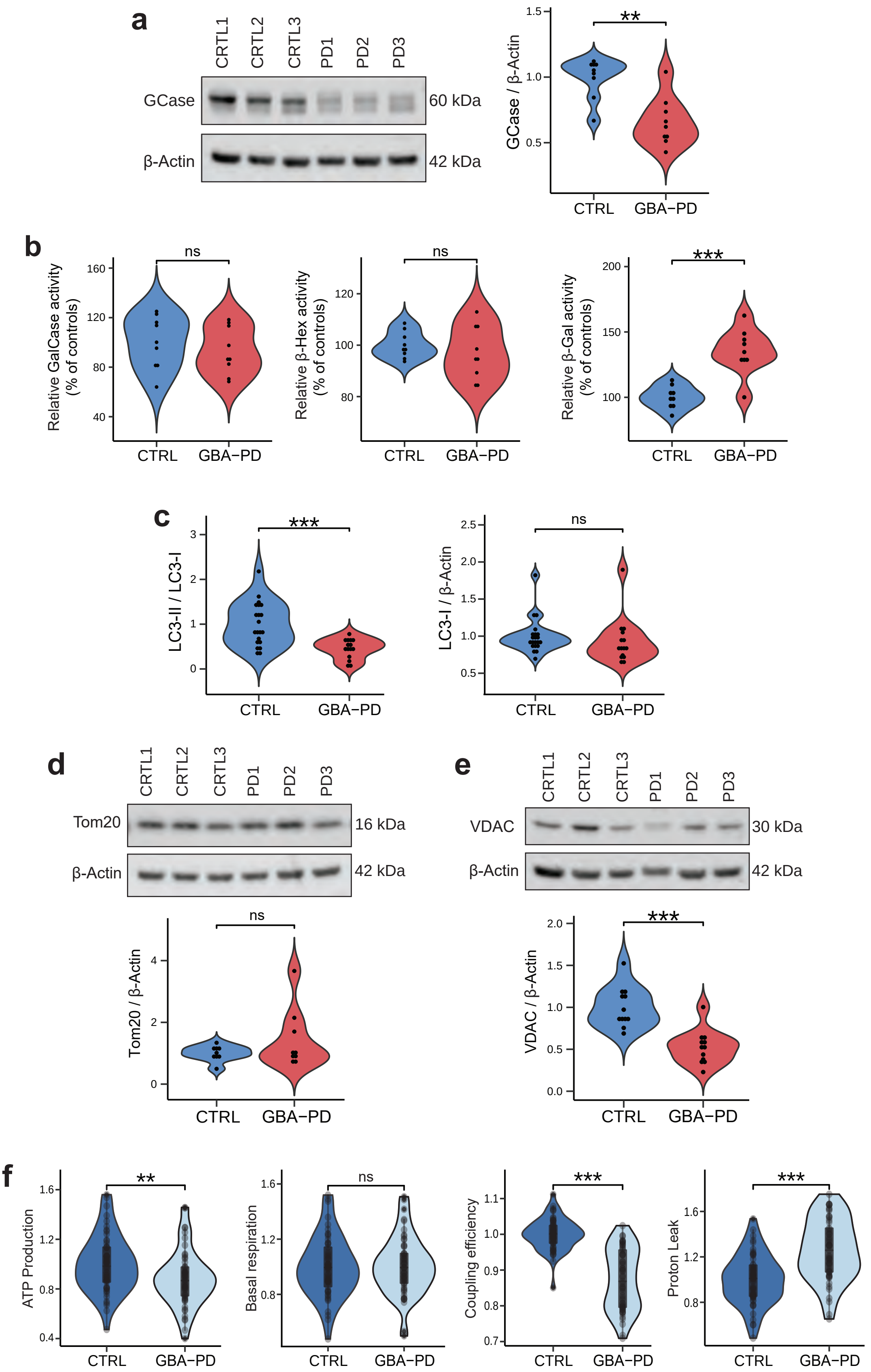

Supplementary Fig. 3. Autophagic and mitochondrial phenotypes in GBA-PD organoids.

a) GCase protein levels are significantly decreased at DIV60. Representative western blot analysis and respective quantification. The data represent a summary of at least three independent differentiation experiments, normalized to the average of controls per organoid batch. Wilcoxon T-test.; **$p < 0.01$.
b) Relative activities of lysosomal hydrolases β-galactocerebrosidase (GalCase), β-hexosaminidase (β-Hex) and β-galactosidase (β-Gal) in differentiated MO cultures. The data represent a summary of three independent differentiation experiments per cell line at DIV30. Values are normalized to the average of controls per experiment. Wilcoxon T-test.; ***$p < 0.001$.
c) Western blot quantification of LC3-II/LC3-I ratio and LC3-I protein at DIV30. Data represents a summary of five independent experiments, normalized to the mean of controls per batch. Wilcoxon T-test.; ***$p < 0.001$.
d) - e) Western blot analysis showing levels of mitochondrial proteins Tom20 and VDAC at DIV30. The data represent a summary of at least three independent differentiation experiments, normalized to the average of controls per organoid batch. Wilcoxon T-test.; ***$p < 0.001$.
f) Quantification of basal and ATP-linked mitochondrial respiration along with coupling efficiency and proton leak. Data are normalized to organoid size (area). The data represent a summary of three independent experiments for all cell lines, normalized to the mean of controls per batch. Wilcoxon T-test.; **$p < 0.01$, ***$p < 0.001$.

Supplementary Fig. 4

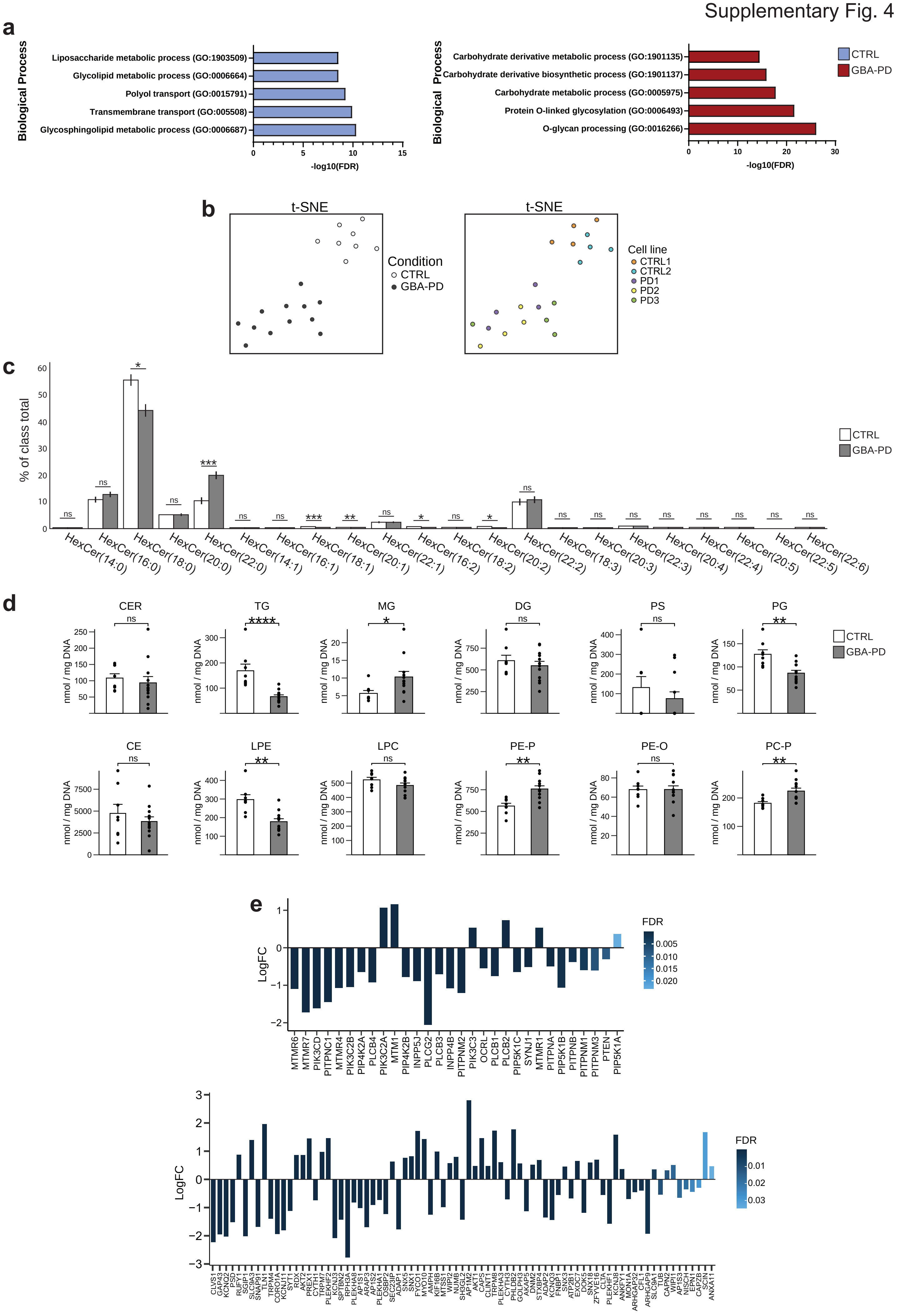

Supplementary Fig. 4. Metabolic modeling of GBA-N370S and wild-type organoids and lipidomics analysis.

a) Gene enrichment analysis of genes found exclusively in control models (blue) and in GBA-PD models (red).
b) t-SNE scatter plots of the complete lipidomics dataset showing a clear clustering of patients (PD1, PD2 and PD3) and controls (CTRL1 and CTRL2).
c) Different hexosylceramide (HexCer) species measured by HILIC LC-MS/MS DIV30 organoids. Data represent a summary of four independent organoid differentiations, FDR adjusted p-values were calculated using the Benjamini–Hochberg procedure. *$p < 0.05$, **$p < 0.01$, ***$p < 0.001$. Error bars represent standard error of the mean.
d) HILIC LC-MS/MS based lipidomic analysis of different lipid classes of DIV30 organoids. ceramides (CER), triacylglycerides (TG), monoacylglycerides (MG), diacylglycerides (DG), phosphatidylserine (PS), phosphatidylglycerol (PG), cholesterol esters (CE), lysophosphatidylethanolamine (LPE), lysophosphatidylcholine (LPC), 1-alkenyl,2-acylphosphatidylethanolamines (PE-P), 1-alkyl,2-acylphosphatidylethanolamines (PE-O), 1-alkenyl,2-acylphosphatidylcholine (PC-P). Data represent a summary of four independent organoid differentiations, Mann-Whitney U test. *$p < 0.05$, **$p < 0.01$, ***$p < 0.001$ ****$p < 0.0001$. Error bars represent standard error of the mean.
e) Expression of phosphoinositide signaling genes in DIV30 hMOs. Top panel shows the DEGs of phospholipases, lipid transfer proteins, kinases and phosphatases. Bottom panel shows the DEGs of phosphoinositide binding proteins. Data is expressed as the logFC of the genes in the comparison patient-derived organoids vs controls.

Supplementary Fig. 5

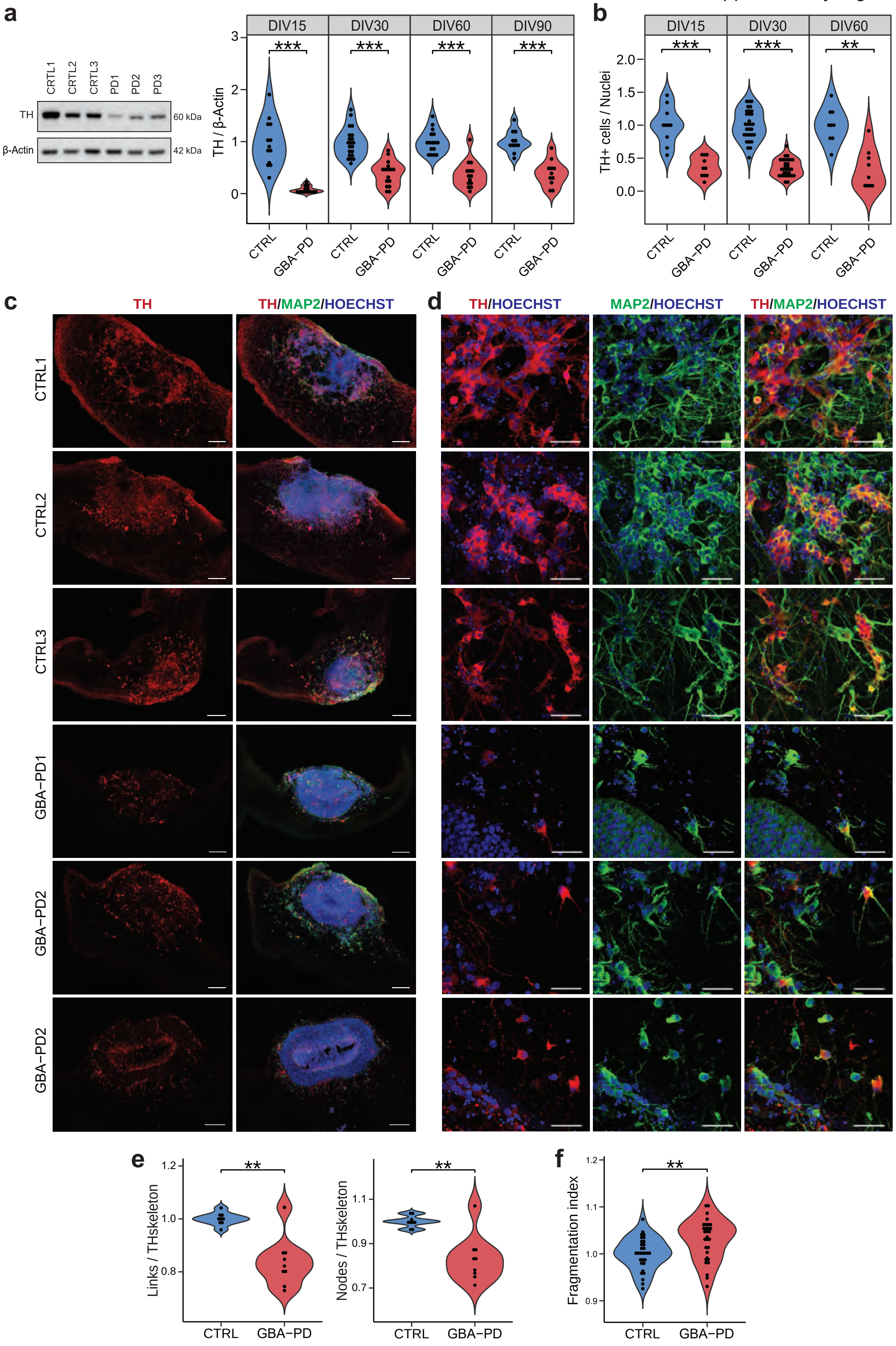

Supplementary Fig. 5. Impaired dopaminergic neuronal differentiation.

a) - b) Quantification dopaminergic neurons levels at DIV15, DIV30, DIV60 and DIV90 by western blot analysis and automated high content image analysis and representative western blot image at DIV15. β-Actin is the same as in Fig. S6c as all three markers (TH, TUJ1 and β-Actin) were blotted in the same membrane. Experiments repeated at least three times. Values are normalized to the mean of controls per organoid batch. Wilcoxon T-test.; *$p < 0.05$, **$p < 0.01$, ***$p < 0.001$.

c) - d) Representative images of DIV30 midbrain organoids sections for all six cell lines stained for TH (red), MAP2 (green), nuclei (blue)) acquired at 20x (C) and 40x (D) (scalebar 200 µm and 50 µm, respectively). Images shown from CTRL1 and PD1 are the same as in Fig. 4c-d.

e) Differences in neurite branching become more significant at DIV60, measured by the number of nodes (branching points) and links (branches) extracted from the skeletonization of TH mask by the algorithm used for image analysis. Values are normalized to the mean of controls per organoid batch. Wilcoxon T-test.; **$p < 0.01$.

f) Higher fragmentation of TH+ neurites in organoids from GBA-PD lines. Fragmentation index is calculated by the image analysis script by computing the surface to volume ratio of the TH mask. Values are normalized to the mean of controls per organoid batch. Wilcoxon T-test.; *$p < 0.05$.

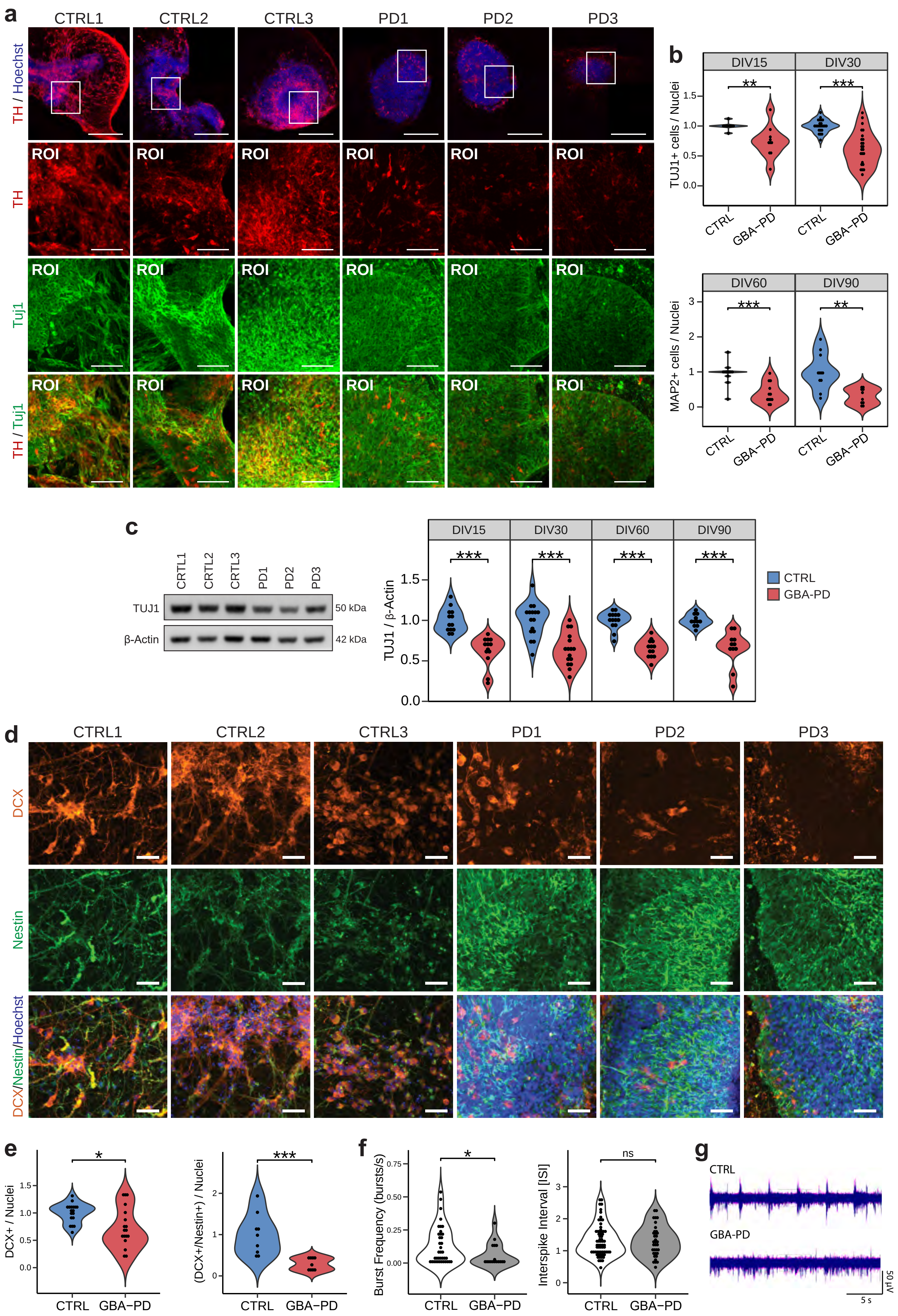
a
CTRL1
CTRL2
CTRL3
PD1
PD2
PD3
TH / Hoechst
ROI
TH
Tuj1
TH / Tuj1
b
DIV15
DIV30
TUJ1+ cells / Nuclei
CTRL
GBA-PD
DIV60
DIV90
MAP2+ cells / Nuclei
c
CRTL1
CRTL2
CRTL3
PD1
PD2
PD3
TUJ1
β-Actin
50 kDa
42 kDa
TUJ1 / β-Actin
CTRL
GBA-PD
d
DCX
Nestin
DCX/Nestin/Hoechst
e
DCX+ / Nuclei
(DCX+/Nestin+) / Nuclei
f
Burst Frequency (bursts/s)
Interspike Interval [ISI]
ns
g
CTRL
GBA-PD
5 s
50 µV

Supplementary Fig. 6. Impaired general neurogenesis.

a) Representative immunofluorescence images of DIV15 organoids expressing TH (red) and Tuj1 (green) with the respective region of interest (ROI). Nuclei were counterstained with Hoechst (blue) (scalebar 200 µm and 50 µm, respectively).
b) Quantification of neuronal content of midbrain organoids showing decreased general neuronal population at every timepoint, TUJ1 was used as an early neuronal marker at DIV15 and DIV30 (top) and MAP2 was a used as a mature neuronal marker at DIV60 and DIV90 (bottom). Data represent a summary of at least 3 independent organoid differentiations, normalized to the mean of controls per experiment. Wilcoxon T-test.; **p < 0.01, ***p < 0.001.
c) Quantification of the neuronal marker TUJ1 at DIV15, DIV30, DIV60 and DIV90 and representative western blot image at DIV15. β-Actin is the same as in Fig. S5a as all three markers (TH, TUJ1 and β-Actin) were blotted in the same membrane. The data represent a summary of at least three independent differentiations, normalized to mean of controls per experiment. Wilcoxon T-test.; ***p < 0.001.
d) Representative images of DIV30 organoid sections stained with the early neuronal marker DCX (orange) and the neural stem cell marker Nestin (green). Nuclei were counterstained with Hoechst (blue) (scalebar 50 µm).
e) Immunofluorescence high content image analysis of DCX (right) at DIV30 normalized to the total amount of cells (nuclei) and the proportion of cells expressing both Nestin and DCX (left), normalized to the total amount of cells (nuclei). The data represent a summary of three independent differentiation experiments per line. Values are normalized to the mean of controls per organoid batch. Wilcoxon T-test.; *p < 0.05.
f) MEA measurements of burst frequency and interspike interval at DIV15. Wilcoxon T-test.; *p < 0.05.
g) Representative raw recordings traces from individual electrodes showing spontaneous activity of firing neurons in CTRL1 and PD1 organoids at DIV15.

Supplementary Fig. 7

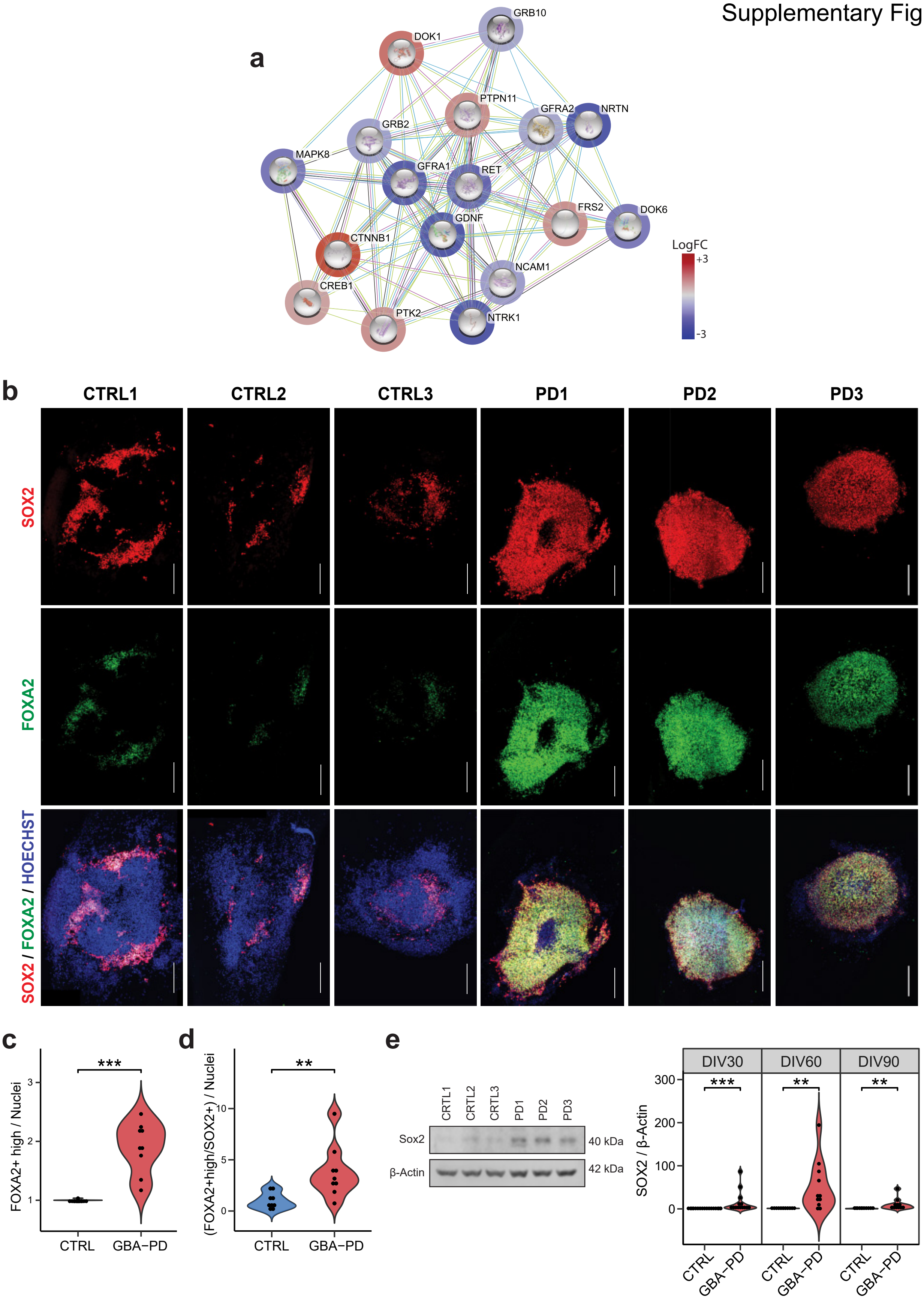

Supplementary Fig. 7. Increased proportion of cells expressing neural stem cell markers.

a) Downregulation of interactive partners of GDNF (obtained from the STRING database) in GBA-N370S organoids based on the differentially expressed genes.
b) Representative confocal images of SOX2+ (red) and FOXA2+ (green) organoid sections at DIV30 Nuclei were counterstained with Hoechst (blue) (scalebar 200 µm).
c) - d) Automated quantification of cells expressing high levels of FOXA2 (left) and FOXA2+/SOX2+ cells (right) in DIV30 organoids normalized to the total amount of cells (nuclei). The data represent a summary of three independent differentiation experiments per line. Wilcoxon T-test.; ***$p < 0.001$.
e) Higher expression of SOX2 protein at DIV30, DIV60 and DIV90 in mutant organoids compared to controls. Representative western blot image at DIV60. Experiments repeated at least three times. Data is normalized to the mean of controls per experiment. Wilcoxon T-test.; **$p < 0.01$, ***$p < 0.001$.

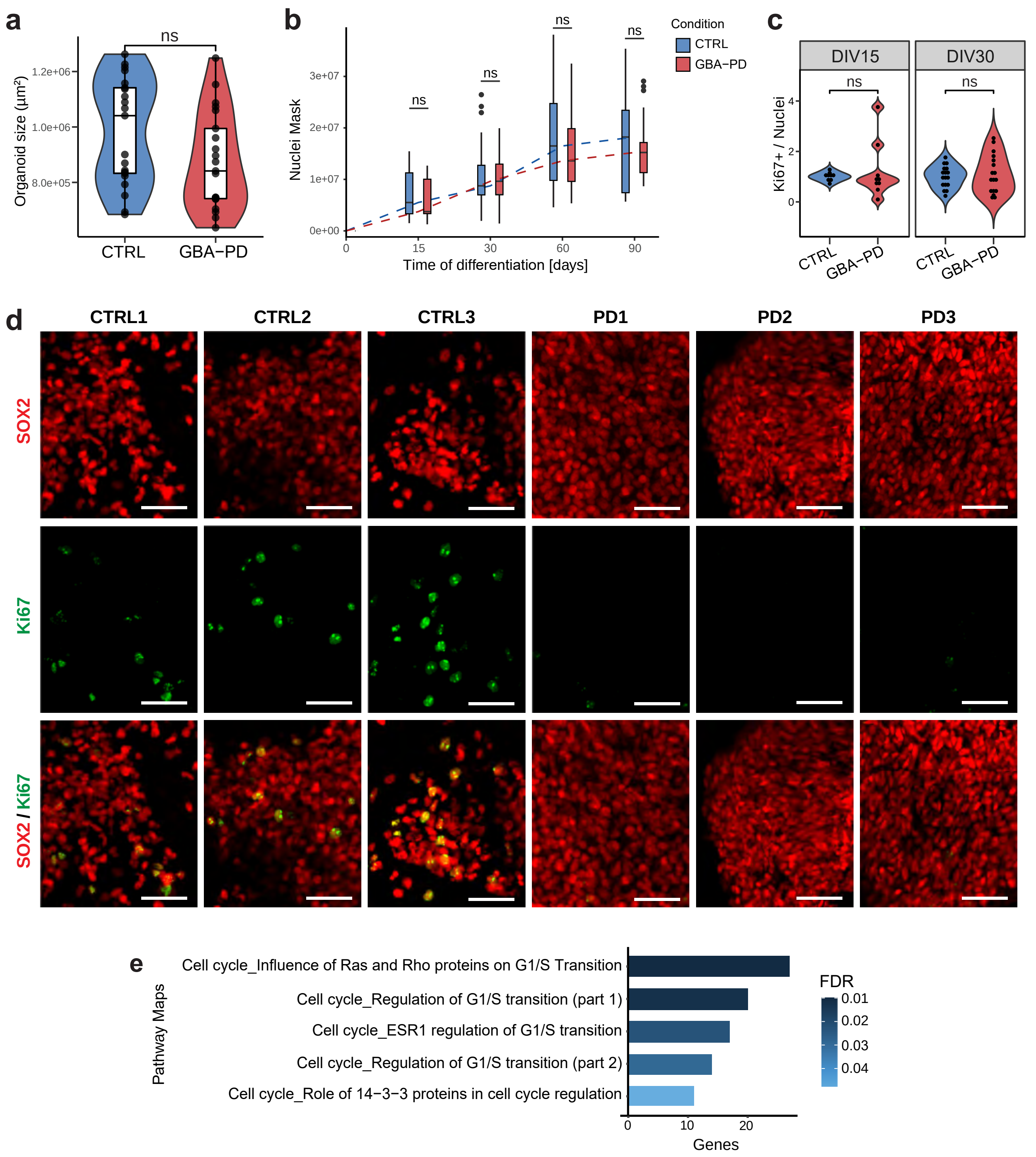

a
ns
Organoid size (µm²)
1.2e+06
1.0e+06
8.0e+05
CTRL
GBA−PD
b
Nuclei Mask
3e+07
2e+07
1e+07
0e+00
0
15
30
60
90
Time of differentiation [days]
ns
Condition
CTRL
GBA−PD
c
DIV15
DIV30
Ki67+ / Nuclei
4
2
0
CTRL
GBA−PD
d
CTRL1
CTRL2
CTRL3
PD1
PD2
PD3
SOX2
Ki67
SOX2 / Ki67
e
Pathway Maps
Cell cycle_Influence of Ras and Rho proteins on G1/S Transition
Cell cycle_Regulation of G1/S transition (part 1)
Cell cycle_ESR1 regulation of G1/S transition
Cell cycle_Regulation of G1/S transition (part 2)
Cell cycle_Role of 14−3−3 proteins in cell cycle regulation
0
10
20
Genes
FDR
0.01
0.02
0.03
0.04

Supplementary Fig. 8. Neural progenitors present lower proliferation capacity in GBA-PD organoids.

a) No differences in organoid size (area) between control and patient-derived MOs at DIV30. Data is normalized to the mean of controls per experiment. Wilcoxon T-test, n=7. In each box plot, the central line represents the median, the lower and upper hinges correspond to the 25th and 75th percentiles.
b) Organoid size during differentiation, measured by the automated pixel count of the nuclear mask (Hoechst). The data represent a summary of at least three independent differentiation experiments per line. Wilcoxon T-test. In each box plot, the central line represents the median, the lower and upper hinges correspond to the 25th and 75th percentiles.
c) Immunofluorescence quantification of cells expressing the proliferative marker Ki67 shows no differences of proliferation between control and mutant conditions at DIV15 and DIV30. Data is normalized to the mean of controls per experiment. Wilcoxon T-test, experiments were repeated at least three times.
d) Representative confocal images of cells expressing SOX2 (red) and Ki67 (green) in DIV30 organoid sections (scalebar 50 µm).
e) Pathway enrichment analysis of differentially expressed genes (DEGs) showing enrichment in pathway maps related to cell cycle regulation (Genego Metacore). Pathways were considered significantly enriched if their P-value adjusted by Benjamini-Hochberg was <0.05.

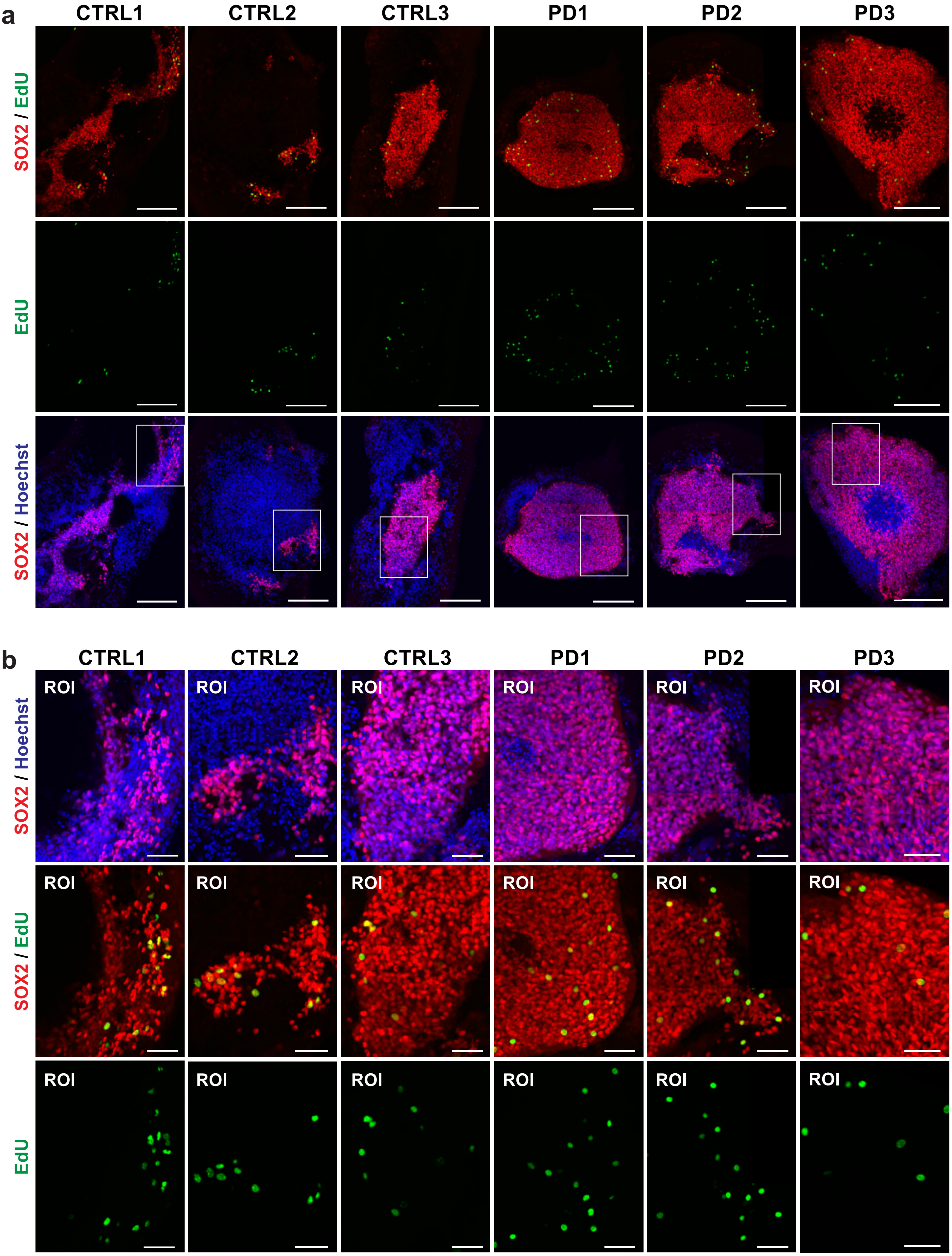
a
CTRL1
CTRL2
CTRL3
PD1
PD2
PD3
SOX2 / EdU
EdU
SOX2 / Hoechst
b
CTRL1
CTRL2
CTRL3
PD1
PD2
PD3
SOX2 / Hoechst
SOX2 / EdU
EdU
ROI

Supplementary Fig. 9. Detection of EdU incorporated into the DNA of after initial pulse.

a) - b) Immunofluorescence detection of Edu incorporation (green) by SOX+ neural progenitors (red) in DIV30 organoids after an 8h pulse (A; scalebar 200 µm) and the respective zoomed region (B; scalebar 50 µm). ROI from organoids CTRL1 and PD1 from panel b are extracted from the same sections as ROI from Fig. 5i. Sections were counterstained with Hoechst to reveal cellular DNA.

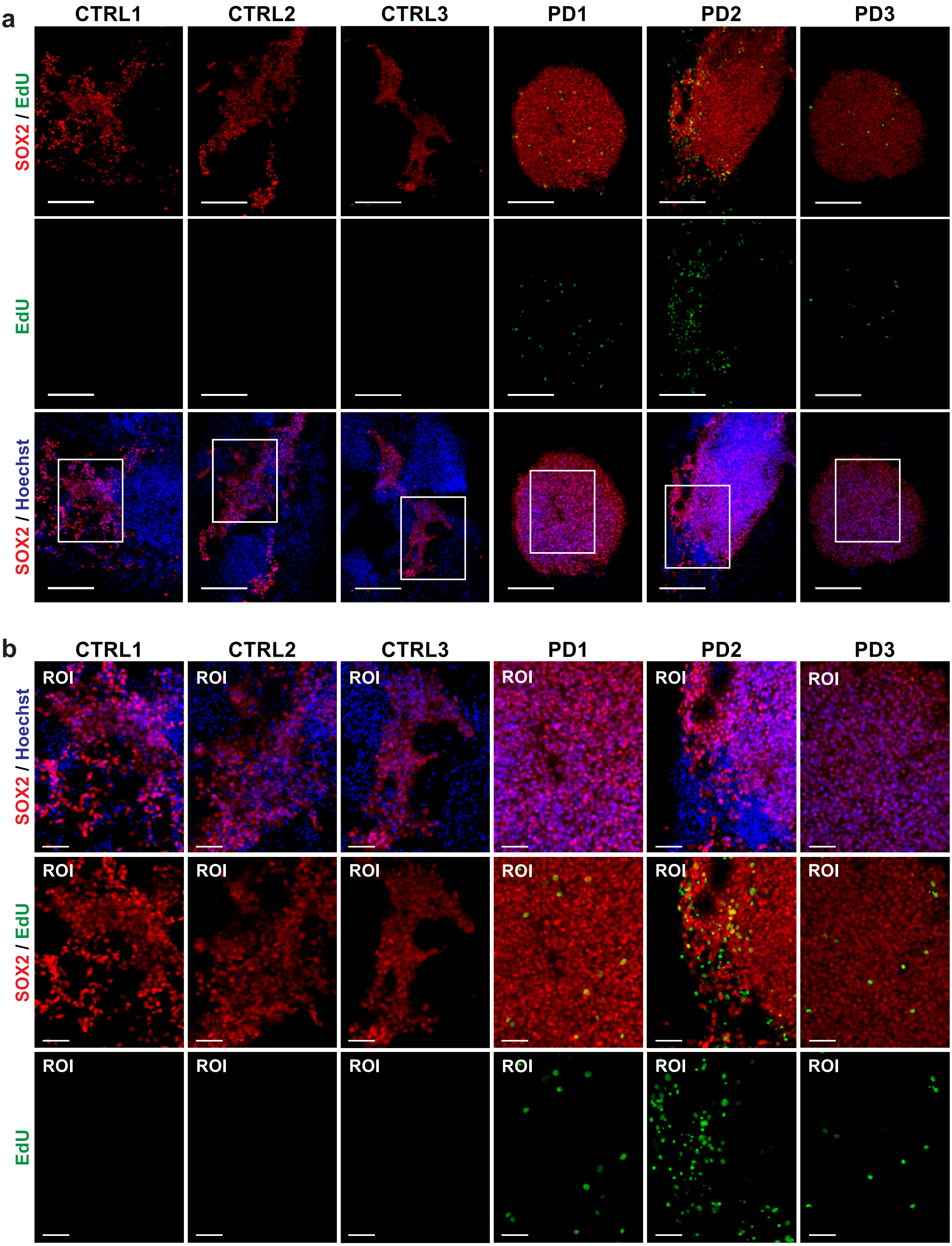
a
CTRL1
CTRL2
CTRL3
PD1
PD2
PD3
SOX2 / EdU
EdU
SOX2 / Hoechst
b
CTRL1
CTRL2
CTRL3
PD1
PD2
PD3
ROI
SOX2 / Hoechst
SOX2 / EdU
EdU

Supplementary Fig. 10. Detection of EdU signal after 7 days from initial pulse.

a) - b) Loss of Edu signal (green) in SOX+ neural progenitors (red) of control MOs after 7 days from the initial pulse (A; scalebar 200 µm) and the respective zoomed region (B; scalebar 50 µm). ROI from organoids CTRL1 and PD1 from panel b are extracted from the same sections as ROI from Fig. 5i. Sections correspond to organoids at DIV37 and were counterstained with Hoechst to reveal cellular DNA.

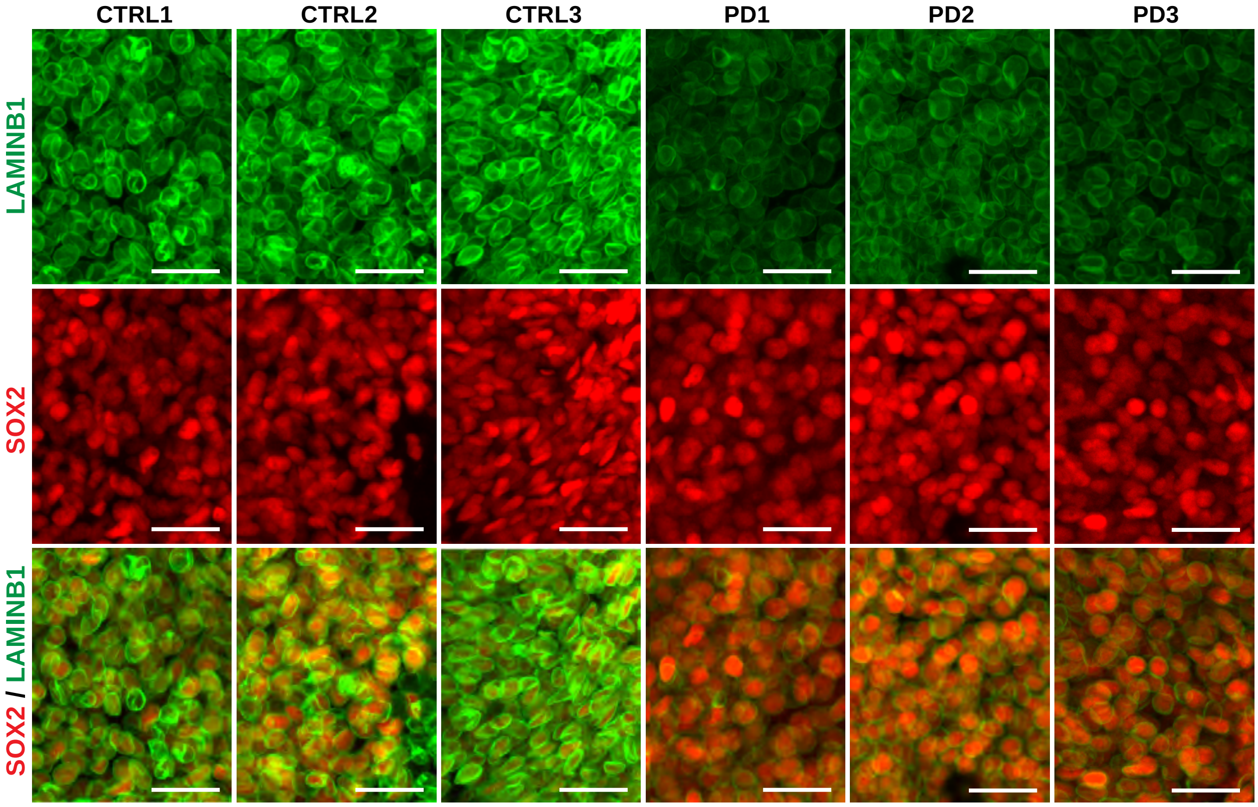
CTRL1
CTRL2
CTRL3
PD1
PD2
PD3
LAMINB1
SOX2
SOX2 / LAMINB1

Supplementary Fig. 11. Expression of senescence-associated markers – LAMINB1.

Representative confocal images of cells expressing SOX2 (red) and LAMINB1 (green) in DIV30 organoid sections (scalebar 25 µm).

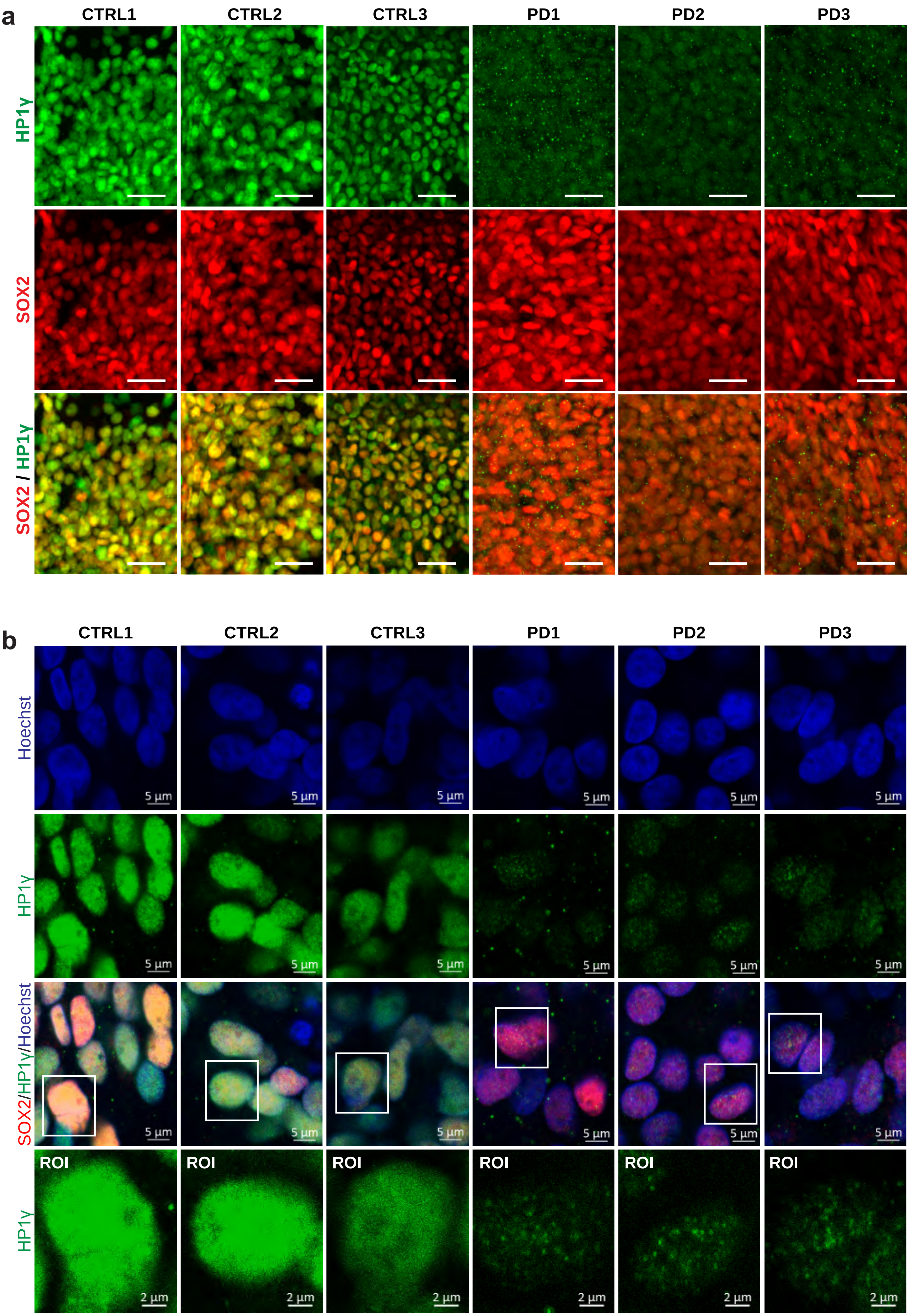
a
CTRL1
CTRL2
CTRL3
PD1
PD2
PD3
HP1γ
SOX2
SOX2 / HP1γ
b
CTRL1
CTRL2
CTRL3
PD1
PD2
PD3
Hoechst
HP1γ
SOX2/HP1γ/Hoechst
ROI
5 µm
2 µm

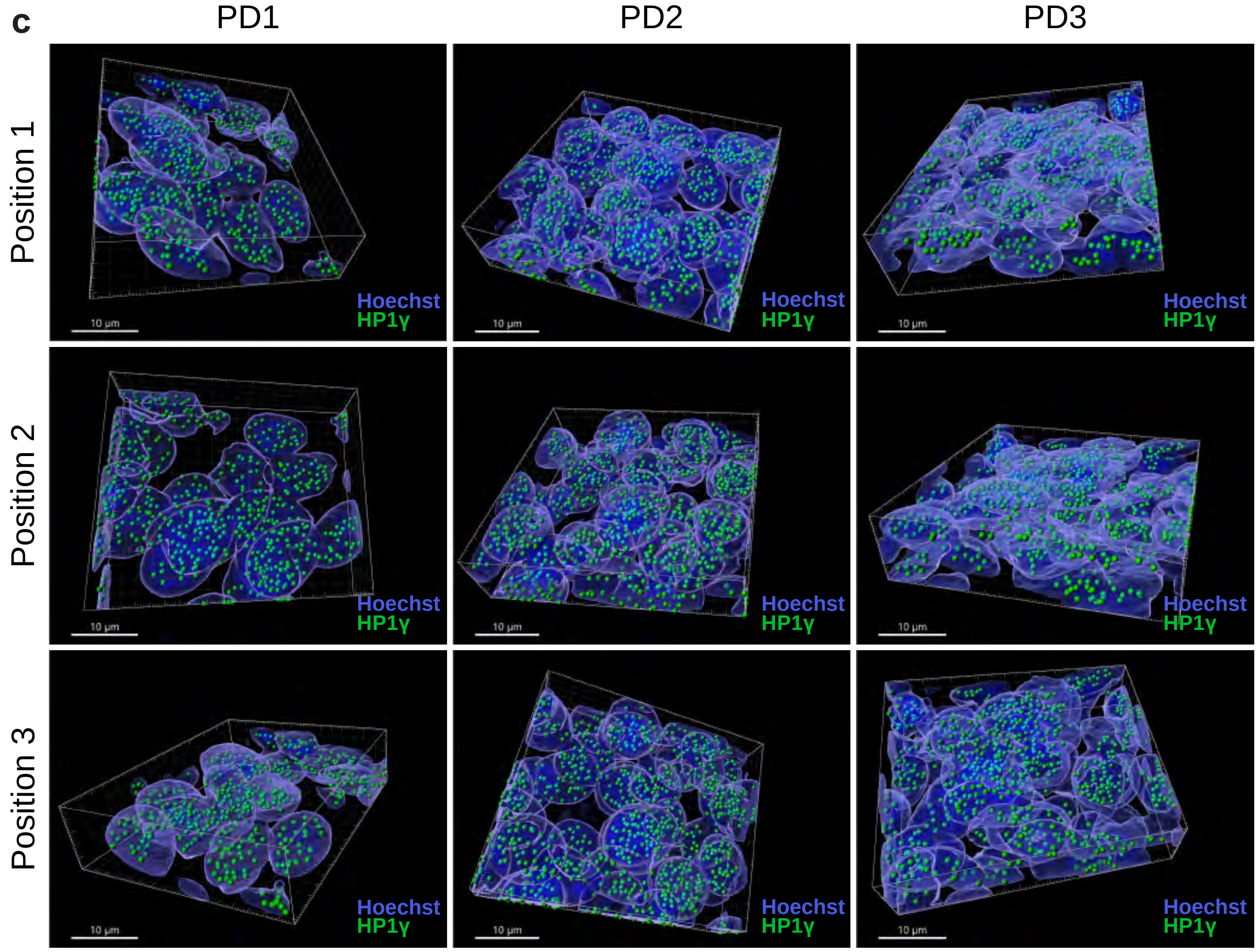
c
PD1
PD2
PD3
Position 1
Position 2
Position 3
Hoechst
HP1γ
10 µm

Supplementary Fig. 12. Expression of senescence-associated markers – HP1γ.

a) Representative confocal images of cells expressing SOX2 (red) and HPIγ (green) in DIV30 organoid sections. Images acquired at 20x magnification (scalebar 25 µm).
b) Presence of dotted pattern of HPIγ (green) in DIV30 GBA-PD organoids. Sox2 (red), Hoechst (blue). Images acquired at 60x magnification with their respective region of interest (ROI) (scalebar 5 µm and 2 µm, respectively). ROI from CTRL2 and PD2 are extracted from the same organoid sections as Fig. 6c.
c) Intranuclear dotted signal of HPIγ (green) in DIV30 GBA-PD organoids identified using the "create spots" algorithm in Imaris. Nuclei were counterstained with Hoechst (blue) and identified using the "surfaces" algorithm (scalebar 10 µm).

Supplementary Fig. 13

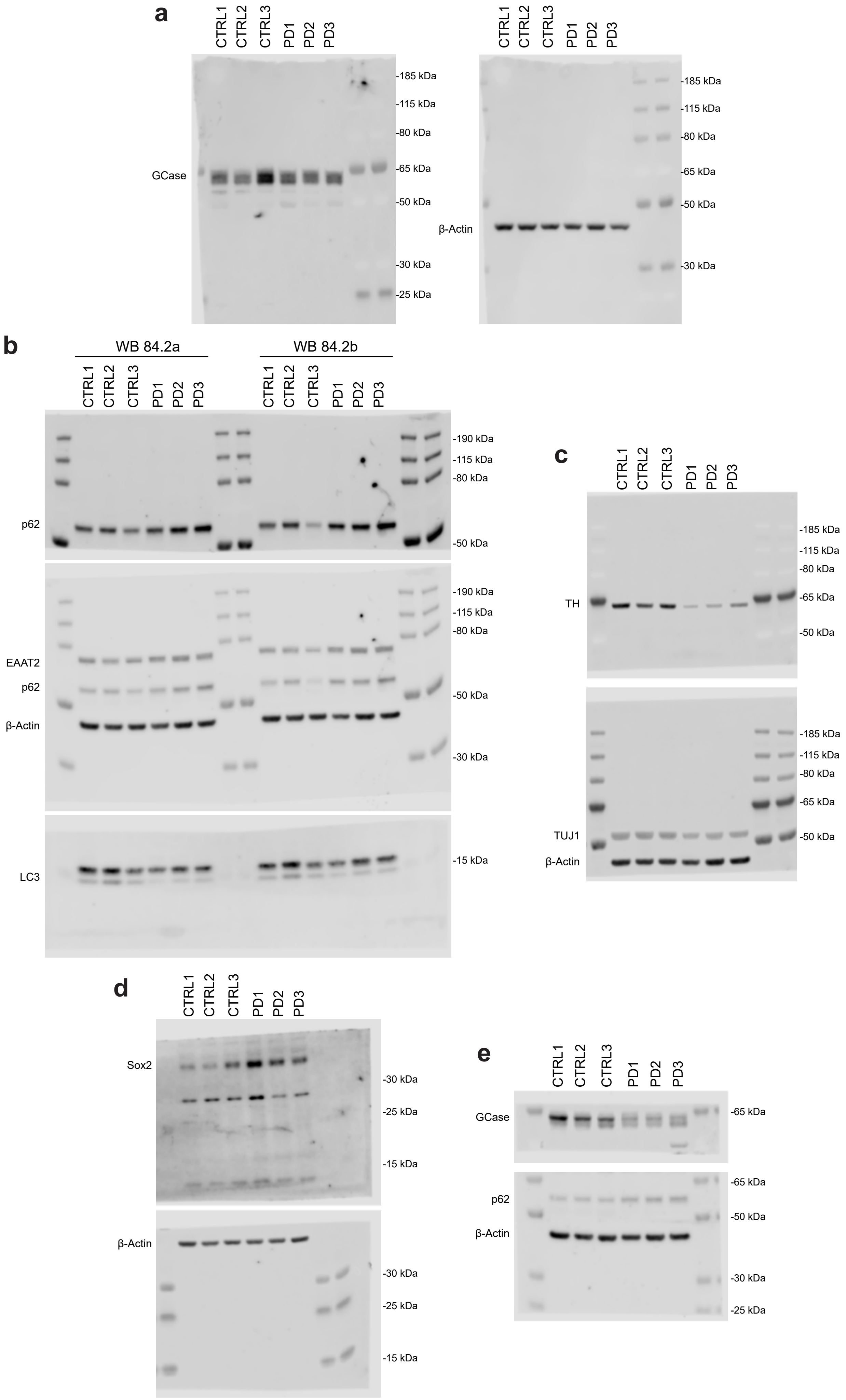

Supplementary Fig. 13

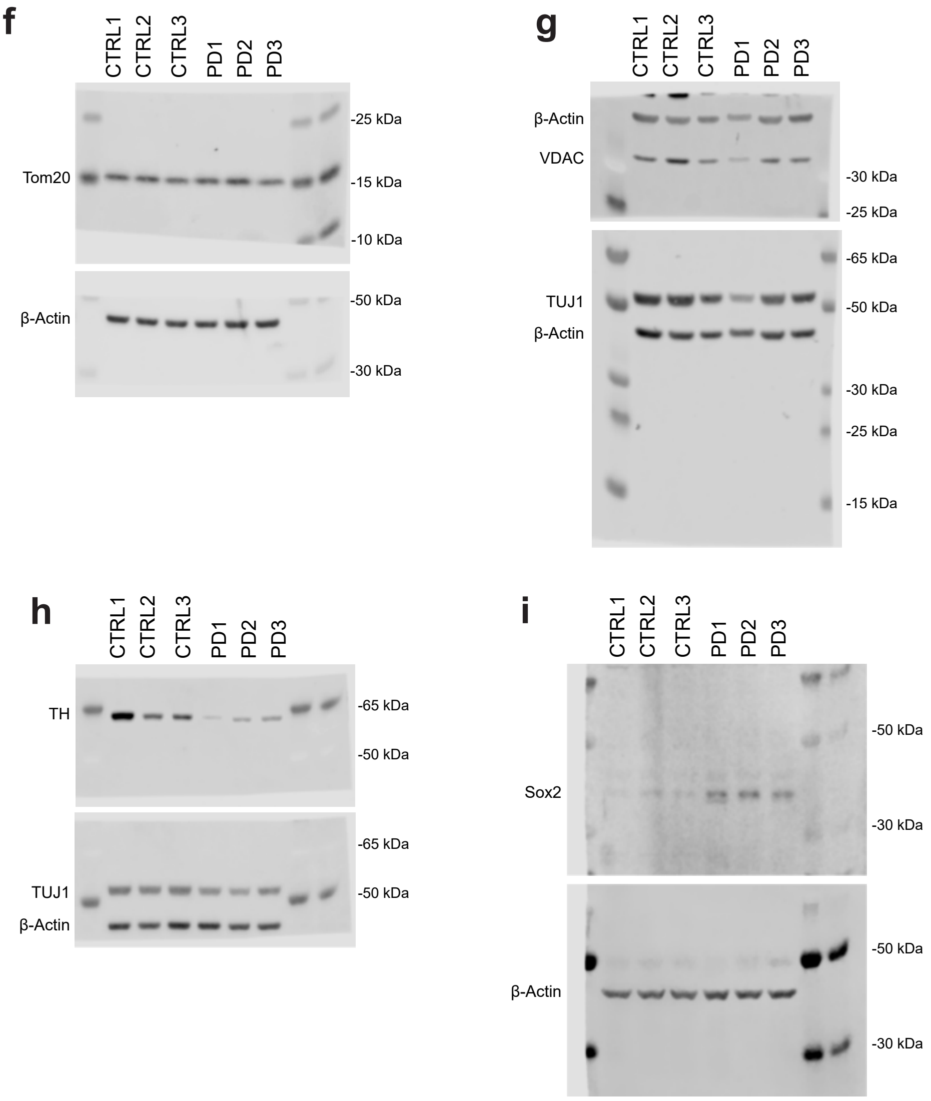

Supplementary Fig. 13. Full length Western blots.

a) Original membrane shown in Fig.1 panel c, blotted for GCase (left) and β-Actin (right).
b) Original membrane shown in Fig.1 panel e (WB84.2b) and f (WB84.2a), blotted for p62, EEAT2, LC3 and β-Actin.
c) Original membrane shown in Fig.4 panel b, blotted for TH (top), TUJ1 and β-Actin (bottom).
d) Original membrane shown in Fig.5 panel d, blotted for Sox2 (top) and β-Actin (bottom).
e) Original membrane shown in Supplementary Fig.3 panel a, blotted for GCase (top), p62 and β-Actin (bottom).
f) Original membrane shown in Supplementary Fig.3 panel d, blotted for Tom20 (top) and β-Actin (bottom).
g) Original membrane shown in Supplementary Fig.3 panel e, blotted for VDAC, TUJ1 and β-Actin.
h) Original membrane shown in Supplementary Fig.5 panel a (TH) and Supplementary Fig.6 panel c (TUJ1) blotted for TH (top), TUJ1 and β-Actin (bottom).
i) Original membrane shown in Supplementary Fig.7 panel e, blotted for Sox2 (top) and β-Actin (bottom).

Supplementary Table 1. Human iPSC used in this study

ID	Diagnosis	Genotype	Age of Biopsy	Age of Onset	Original IDs
Control-1	Healthy	wt/wt	68	-	3012659-MDPD1
Control-2	Healthy	wt/wt	65	-	2716623-MDPD1
Control-3	Healthy	wt/wt	68	-	68
GBA-PD-1	PD	N370S/wt	69	63	ND31630 Cl5
GBA-PD-2	PD	N370S/wt	55	Unknown	KTI6
GBA-PD-3	PD	N370S/wt	75	Unknown	SGO1

Supplementary Table 2. Primary antibodies used in this study

Antibody	Species	Source	Ref.-No.	RRID	WB Dilution	IF Dilution
β-Actin	Mouse	Cell Signaling	3700	AB_2242334	1:100 000	-
DCX	Guinea Pig	Millipore	AB2253	AB_1586992	-	1:400
FOXA2	Mouse	Santa Cruz	sc-101060	AB_1124660	-	1:250
GBA	Mouse	Abnova	H00002629-M01	AB_1505986	-	1:500
GBA	Rabbit	Sigma	G4171	AB_1078958	1:500	-
HPIg	Rabbit	Cell Signaling	2619S	AB_2070984	-	1:200
Ki67	Mouse	BD Biosciences	550609	AB_393778	-	1:200
LAMP1	Mouse	Abcam	ab25630	AB_470708	-	1:400
LaminB1	Rabbit	Abcam	ab16048	AB_443298		1:200
LC3	Rabbit	MBL	PM036	AB_2274121	1:1000	1:1000
MAP2	Rabbit	Abcam	ab32454	AB_2138147	-	1:100
MAP2	Mouse	Millipore	MAB3418	AB_94856	-	1:200
MAP2	Chicken	Abcam	ab92434	AB_2138147	-	1:1000
Nestin	Mouse	Millipore	MAB5326	AB_2251134	-	1:100
Pax6	Rabbit	Covance	PRB-278P	AB_291612	-	1:300
P62 (SQSTM1)	Mouse	Abcam	ab56416	AB_945626	1:500	-
Sox2	Goat	R&D Systems	AF2018	AB_355110	-	1:100
Sox2	Rabbit	Abcam	ab97959	AB_2341193	1:500	-
TH	Rabbit	Santa Cruz	sc-14007	AB_671397	1:1000	-
TH	Rabbit	Abcam	ab112	AB_297840	-	1:1000
Tom20	Mouse	Santa Cruz	sc-17764	AB_628381	1:500	-
TUJ1	Chicken	Millipore	AB9354	AB_570918	-	1:600
TUJ1	Mouse	BioLegend	801201	AB_2313773	1: 50 000	-
VDAC	Rabbit	Cell Signaling	4661	AB_10557420	1:1000	-

Supplementary Table 3. Metabolic model composition per cell line

Model	Genes	Reactions	Metabolites
CTRL1	1706	3648	2685
CTRL2	1716	3454	2570
PD1	1635	3990	2917
PD2	1672	4119	2997

Supplementary Table 4. Reactions related to dopamine metabolism present only in control metabolic models

Reaction ID	Pathway	Reaction
'DOPAENT4tc'	'Transport, extracellular'	'h[e] + dopa[e] <=> h[c] + dopa[c] '
'r2494'	'Transport, extracellular'	'hco3[e] + dopa[c] + HC02199[e] -> hco3[c] + dopa[e] + HC02199[c] '
'r2495'	'Transport, extracellular'	'hco3[e] + dopa[c] + HC02200[e] -> hco3[c] + dopa[e] + HC02200[c] '
'r2496'	'Transport, extracellular'	'hco3[e] + dopa[c] + HC02201[e] -> hco3[c] + dopa[e] + HC02201[c] '

Supplementary Table 5. Number of reactions per subsystem present only in control models and GBA-PD models, respectively.

Pathway	Rxn number in CTRL models	Pathway	Rxn number in GBA-PD models
Transport, extracellular	116	Transport, extracellular	292
Fatty acid oxidation	75	Exchange/demand reaction	275
Exchange/demand reaction	55	Peptide metabolism	242
Sphingolipid metabolism	26	Fatty acid oxidation	60
Transport, mitochondrial	17	Glycerophospholipid metabolism	19
Valine, leucine, and isoleucine metabolism	9	Nucleotide interconversion	16
Transport, golgi apparatus	9	Transport, mitochondrial	13
Miscellaneous	9	Keratan sulfate degradation	13
Transport, endoplasmic reticular	8	Keratan sulfate synthesis	11
Phosphatidylinositol phosphate metabolism	7	Transport, peroxisomal	9
NAD metabolism	6	Transport, endoplasmic reticular	9
Bile acid synthesis	5	Drug metabolism	7
Glycerophospholipid metabolism	5	Tryptophan metabolism	6
Transport, nuclear	5	Transport, lysosomal	6
Transport, peroxisomal	5	Folate metabolism	6
Glycosphingolipid metabolism	5	Eicosanoid metabolism	5
Folate metabolism	5	Transport, nuclear	4
Tyrosine metabolism	4	Chondroitin sulfate degradation	4
Transport, lysosomal	4	Pentose phosphate pathway	4
Nucleotide interconversion	4	Lysine metabolism	4
Steroid metabolism	4	Phosphatidylinositol phosphate metabolism	4
Urea cycle	4	Glycine, serine, alanine, and threonine metabolism	3
Vitamin B6 metabolism	4	Pyrimidine catabolism	3
C5-branched dibasic acid metabolism	4	O-glycan metabolism	3
Tryptophan metabolism	3	Glycosphingolipid metabolism	3
N-glycan synthesis	3	Inositol phosphate metabolism	3
Fructose and mannose metabolism	3	Bile acid synthesis	3
Glyoxylate and dicarboxylate metabolism	3	Aminosugar metabolism	2
Pentose phosphate pathway	3	Blood group synthesis	2
Phenylalanine metabolism	3	Sphingolipid metabolism	2
Fatty acid synthesis	3	Transport, golgi apparatus	2
Eicosanoid metabolism	3	Pyruvate metabolism	2
Butanoate metabolism	2	Phenylalanine metabolism	2
Propanoate metabolism	2	Purine catabolism	2
Aminosugar metabolism	2	Chondroitin synthesis	2

Cholesterol metabolism	2	Propanoate metabolism	1
Androgen and estrogen synthesis and metabolism	2	Glutathione metabolism	1
N-glycan metabolism	2	ROS detoxification	1
Inositol phosphate metabolism	2	Cholesterol metabolism	1
Purine catabolism	2	Purine synthesis	1
Methionine and cysteine metabolism	2	NAD metabolism	1
Ubiquinone synthesis	1	Pyrimidine synthesis	1
Glycolysis/gluconeogenesis	1	Starch and sucrose metabolism	1
Lysine metabolism	1	Heme degradation	1
Vitamin C metabolism	1	Glycolysis/gluconeogenesis	1
Oxidative phosphorylation	1		
Leukotriene metabolism	1		
Glycine, serine, alanine, and threonine metabolism	1		
Pyrimidine catabolism	1		
Vitamin B2 metabolism	1		
Triacylglycerol synthesis	1		
Chondroitin synthesis	1		
Galactose metabolism	1		
