# Supplementary material for: Impaired neuron differentiation in GBA-associated Parkinson’s disease is linked to cell cycle defects in organoids
Source: NPJ Parkinsons Dis. 2023 Dec 18;9:166. doi: 10.1038/s41531-023-00616-8 (PMC10728202; doi:10.1038/s41531-023-00616-8)
Supplement: Supplementary file 2 — Reporting summary [file 41531_2023_616_MOESM2_ESM.pdf]

nature portfolio

NPJPARKD-01806R1
Corresponding author(s): Jens C. Schwamborn
Last updated by author(s): Sep 23, 2023

Reporting Summary

Nature Portfolio wishes to improve the reproducibility of the work that we publish. This form provides structure for consistency and transparency in reporting. For further information on Nature Portfolio policies, see our Editorial Policies and the Editorial Policy Checklist.

Statistics

For all statistical analyses, confirm that the following items are present in the figure legend, table legend, main text, or Methods section.

n/a	Confirmed	
☐	☒	The exact sample size (*n*) for each experimental group/condition, given as a discrete number and unit of measurement
☐	☒	A statement on whether measurements were taken from distinct samples or whether the same sample was measured repeatedly
☐	☒	The statistical test(s) used AND whether they are one- or two-sided<br>*Only common tests should be described solely by name; describe more complex techniques in the Methods section.*
☒	☐	A description of all covariates tested
☐	☒	A description of any assumptions or corrections, such as tests of normality and adjustment for multiple comparisons
☒	☐	A full description of the statistical parameters including central tendency (e.g. means) or other basic estimates (e.g. regression coefficient) AND variation (e.g. standard deviation) or associated estimates of uncertainty (e.g. confidence intervals)
☒	☐	For null hypothesis testing, the test statistic (e.g. *F*, *t*, *r*) with confidence intervals, effect sizes, degrees of freedom and *P* value noted<br>*Give P values as exact values whenever suitable.*
☒	☐	For Bayesian analysis, information on the choice of priors and Markov chain Monte Carlo settings
☒	☐	For hierarchical and complex designs, identification of the appropriate level for tests and full reporting of outcomes
☒	☐	Estimates of effect sizes (e.g. Cohen's *d*, Pearson's *r*), indicating how they were calculated

Our web collection on statistics for biologists contains articles on many of the points above.

Software and code

Policy information about availability of computer code

Data collection	Matlab 2019/2021 was used to extract all image analysis data and the code is available at https://doi.org/10.17881/xfh3-a153. AxIS software (v.2.1,Axion) was used for multi electrode array recordings.For metabolic modeling, Matlab 2019 was used using COBRA toolbox and FASTCORMICS pipeline previously published. For unique gene functional classification, the David enrichment tool was used, which is freely available online at https://david.ncifcrf.gov/home.jsp. For flux variability analysis, IBM_CPLEX solver (version 12.10) was used.
Data analysis	Data analysis with GraphPad Prism 9 and RStudio (R version 4.0.2). FACS data were analyzed using FlowJo software (v.10.7.2). Western blot data was analyzed using Image studio Lite (version 5.2).

For manuscripts utilizing custom algorithms or software that are central to the research but not yet described in published literature, software must be made available to editors and reviewers. We strongly encourage code deposition in a community repository (e.g. GitHub). See the Nature Portfolio guidelines for submitting code & software for further information.

Data

Policy information about availability of data

All manuscripts must include a data availability statement. This statement should provide the following information, where applicable:

- Accession codes, unique identifiers, or web links for publicly available datasets
- A description of any restrictions on data availability
- For clinical datasets or third party data, please ensure that the statement adheres to our policy

The data is available under a unique DOI: https://doi.org/10.17881/xfh3-a153. RNA sequencing data is available on Gene Expression Omnibus (GEO) under the accession code GSE208784. No restrictions on data availability and for scripts we applied a ApacheV2 license.

Research involving human participants, their data, or biological material

Policy information about studies with human participants or human data. See also policy information about sex, gender (identity/presentation), and sexual orientation and race, ethnicity and racism.

Reporting on sex and gender	In this study we used human induced-pluripotent stem cells from three GBA-Parkinson's disease patients and three age matched healthy individuals.
Reporting on race, ethnicity, or other socially relevant groupings	*Please specify the socially constructed or socially relevant categorization variable(s) used in your manuscript and explain why they were used. Please note that such variables should not be used as proxies for other socially constructed/relevant variables (for example, race or ethnicity should not be used as a proxy for socioeconomic status).*<br>*Provide clear definitions of the relevant terms used, how they were provided (by the participants/respondents, the researchers, or third parties), and the method(s) used to classify people into the different categories (e.g. self-report, census or administrative data, social media data, etc.)*<br>*Please provide details about how you controlled for confounding variables in your analyses.*
Population characteristics	*Describe the covariate-relevant population characteristics of the human research participants (e.g. age, genotypic information, past and current diagnosis and treatment categories). If you filled out the behavioural & social sciences study design questions and have nothing to add here, write "See above."*
Recruitment	*Describe how participants were recruited. Outline any potential self-selection bias or other biases that may be present and how these are likely to impact results.*
Ethics oversight	The use of existing iPSC lines obtained from previous studies was approved by the local ethical committee (Comité National d'Ethique de Recherche). Cell lines used in this study are summarized in Supplementary Table 1.

Note that full information on the approval of the study protocol must also be provided in the manuscript.

Field-specific reporting

Please select the one below that is the best fit for your research. If you are not sure, read the appropriate sections before making your selection.

☒ Life sciences ☐ Behavioural & social sciences ☐ Ecological, evolutionary & environmental sciences

For a reference copy of the document with all sections, see nature.com/documents/nr-reporting-summary-flat.pdf

Life sciences study design

All studies must disclose on these points even when the disclosure is negative.

Sample size	For each group (healthy and PD) three cell lines were used and a minimum of three organoid batches for all cell lines was generated for each experiment.
Data exclusions	In the Seahorse mitochondrial assay data was excluded in case of failed response to the drugs. MEA electrode recordings were excluded based on IQR 1.5 method.
Replication	Reproducibility is confirmed by obtaining consistent results from different organoid generations.
Randomization	N/A
Blinding	The blinding was not done, experiments for both conditions (healthy VS GBA-PD) were always done at the same, with the same reagents and analysis settings to ensure the accuracy.

Reporting for specific materials, systems and methods

We require information from authors about some types of materials, experimental systems and methods used in many studies. Here, indicate whether each material, system or method listed is relevant to your study. If you are not sure if a list item applies to your research, read the appropriate section before selecting a response.

Materials & experimental systems

n/a	Involved in the study	
☐	☒	Antibodies
☐	☒	Eukaryotic cell lines
☒	☐	Palaeontology and archaeology
☒	☐	Animals and other organisms
☒	☐	Clinical data
☒	☐	Dual use research of concern
☒	☐	Plants

Methods

n/a	Involved in the study	
☒	☐	ChIP-seq
☐	☒	Flow cytometry
☒	☐	MRI-based neuroimaging

Antibodies

Antibodies used	All primary antibodies used in the study are listed and described Table S2.
Validation	RRIDs are specified in Table S2

Eukaryotic cell lines

Policy information about cell lines and Sex and Gender in Research

Cell line source(s)	IBBL / Max Planck Institute, StemBANCC, http://ccr.coriell.org, University College London
Authentication	Cell lines have been karyotyped and characterized by pluripotency marker expression
Mycoplasma contamination	All cell lines were tested negative for Mycoplasma
Commonly misidentified lines (See ICLAC register)	N/A

Flow Cytometry

Plots

Confirm that:

☒ The axis labels state the marker and fluorochrome used (e.g. CD4-FITC).

☒ The axis scales are clearly visible. Include numbers along axes only for bottom left plot of group (a 'group' is an analysis of identical markers).

☒ All plots are contour plots with outliers or pseudocolor plots.

☒ A numerical value for number of cells or percentage (with statistics) is provided.

Methodology

Sample preparation	Organoids were dissociated into single cells using papain and accutase, then fixed with ethanol and stained with propidium iodide. More details can be found in materials & methods
Instrument	Becton Dickinson LSRFortessa
Software	BD FACSDiva Software, FlowJo
Cell population abundance	No cell sorting was done
Gating strategy	First gate used was SSC-H/FSC-H to remove debris. Second gate was FSC-H/FSC-A to select single cells.For cell cycle analysis the Watson Pragmatic algorithm was used.

☐ Tick this box to confirm that a figure exemplifying the gating strategy is provided in the Supplementary Information.
